# The atomic structures of shrimp nodaviruses reveal new dimeric spike structures and particle polymorphism

**DOI:** 10.1038/s42003-019-0311-z

**Published:** 2019-02-20

**Authors:** Nai-Chi Chen, Masato Yoshimura, Naoyuki Miyazaki, Hong-Hsiang Guan, Phimonphan Chuankhayan, Chien-Chih Lin, Shao-Kang Chen, Pei-Ju Lin, Yen-Chieh Huang, Kenji Iwasaki, Atsushi Nakagawa, Sunney I. Chan, Chun-Jung Chen

**Affiliations:** 1 Life Science Group, Scientific Research Division, National Synchrotron Radiation Research Center, Hsinchu, 30076 Taiwan; 2 Institute for Protein Research, Osaka University, Suita, Osaka, 565-0871 Japan; 3 Department of Biotechnology and Bioindustry Sciences, National Cheng Kung University, Tainan, 701 Taiwan; 4 Institute of Bioinformatics and Structural Biology, National Tsing Hua University, Hsinchu, 30043 Taiwan; 5 Institute of Chemistry, Academia Sinica, Nankang, Taipei, 11529 Taiwan; 6 Noyes Laboratory 127-72, California Institute of Technology, Pasadena, 91125 CA USA; 7 Department of Physics, National Tsing Hua University, Hsinchu, 30043 Taiwan; 8 Present Address: Life Science Center for Survival Dynamics, Tsukuba Advanced Research Alliance, University of Tsukuba, Tsukuba, Ibaraki, 305-8577 Japan

## Abstract

Shrimp nodaviruses, including *Penaeus vannamei* (PvNV) and *Macrobrachium rosenbergii* nodaviruses (MrNV), cause white-tail disease in shrimps, with high mortality. The viral capsid structure determines viral assembly and host specificity during infections. Here, we show cryo-EM structures of *T* = 3 and *T* = 1 PvNV-like particles (PvNV-LPs), crystal structures of the protrusion-domains (P-domains) of PvNV and MrNV, and the crystal structure of the ∆N-ARM-PvNV shell-domain (S-domain) in *T* = 1 subviral particles. The capsid protein of PvNV reveals five domains: the P-domain with a new jelly-roll structure forming cuboid-like spikes; the jelly-roll S-domain with two calcium ions; the linker between the S- and P-domains exhibiting new cross and parallel conformations; the N-arm interacting with nucleotides organized along icosahedral two-fold axes; and a disordered region comprising the basic *N*-terminal arginine-rich motif (N-ARM) interacting with RNA. The N-ARM controls *T* = 3 and *T* = 1 assemblies. Increasing the *N*/*C*-termini flexibility leads to particle polymorphism. Linker flexibility may influence the dimeric-spike arrangement.

## Abstract (web summary)

Nai-Chi Chen et al. solved the structures of two shrimp nodaviruses, focusing on the major domains to improve understanding of capsid organization. By combining cryo-EM and x-ray crystallography, the authors were able to observe the structures at a high resolution.

## Introduction

P*enaeus vannamei* (*P. vannamei*) and *Macrobrachium rosenbergii* (*M. rosenbergii*) are cultured prawns of economic importance for 90% of the shrimp-aquaculture industry. In the western hemisphere, *P. vannamei* is the species of choice as it can live in a wide range of salinity from 0 to 40 ppt^1^. *M. rosenbergii* is also a cultured freshwater prawn of economic value worldwide. This species is native to Southeast Asian and is produced in Israel, Japan, Taiwan, some African, Latin American, and Caribbean countries^2^.

A major factor that limits the expansion of shrimp cultures is, however, infectious diseases from viruses, such as *P. vannamei* nodavirus (PvNV) and *M. rosenbergii* nodavirus (MrNV), which have inflicted heavy economic losses^3,4^. The white-tail disease, or the white-muscle disease caused by these shrimp nodaviruses, can infect the larvae, postlarvae and early juvenile stages, reaching to 100% mortality within 6 days after appearance of the first gross signs of syndromes, including a white or milky abdominal muscle^3,5–7^. This white-tail disease causes serious large-scale mortalities during the hatchery and nursery phases of the prawn aquaculture^8,9^.

PvNV and MrNV belong to the family *Nodaviridae*, the important genera comprising two other major types: alphanodavirus, infecting insects; and betanodavirus, infecting fishes^10^. The *Nodaviridae* genome comprises two single-stranded positive-sense RNA that encode the capsid protein for viral capsid assembly, the RNA polymerase for RNA replication, and the B2 protein for host RNA interference suppressor^11–13^. An assemblage of 180 capsid proteins with molecular masses ~37 kDa forms nonenveloped icosahedral *T* = 3 virus particles ~29–35 nm in diameter in the PvNV and MrNV^6,14^. A low-resolution cryo-EM map of a MrNV *T* = 3 virus-like particle indicated that the capsid proteins exist as dimers^15^, differing from those of alphanodaviruses and betanodaviruses^13^. The overall amino-acid sequence of the PvNV capsid protein shares ~45% identity with that of the MrNV. While the shell domains (S-domains) of PvNV and MrNV contain conserved sequences of 62% identity, the protrusion domains (P-domains) and N-arms show low sequence identities of only 17% and 43%, respectively.

In this work, we have determined the cryo-EM structures of *T* = 3 and *T* = 1 PvNV-LPs at atomic resolution, the crystal structures of the P-domains of PvNV and MrNV (PvNVPd and MrNVPd), and the crystal structure of the S-domain of PvNV (ΔN-ARM-PvNVSd) in the *T* = 1 subviral particle (SVP). These structures reveal the molecular details of the *T* = 3 PvNV-LP; and the dimeric conformations of the PvNVPd and MrNVPd with the metal-ion coordination. The P-domains reveal a new structural fold related to the dimer–dimer interactions in the *T* = 3 capsid. The variant residues on the P-domains between PvNV and MrNV might reflect their host specificities. Surprisingly, the *T* = 3 PvNV-LP capsid displays specific organizations of the N-arm and the linker in two types of dimers (referred later as subunit-A/B and C/C dimers) that have never been seen in other viruses. Moreover, the dimeric P-domains, the inherent flexibility of *N*/*C*-termini, and the nucleotide organization provide structural insights into the capsid assembly and the molecular switch responsible for particle polymorphism between *T* = 3 and *T* = 1 architectures. The work combines X-ray crystallography, offering structural details of each domain at high resolution, with the cryo-EM, imaging the entire particle structure and some disordered regions that are otherwise not observed by the X-rays albeit at lower resolution. Given that cryo-EM is still behind X-ray crystallography in terms of atomic resolution, this combined approach provides a powerful complementary integrated strategy for the determination of highly complex structures.

## Results

### The overall structure of *T* = 3 PvNV

The overall flow chart for the structural characterizations of the PvNV is depicted in Supplementary Fig. 1. All the viral particles and capsid–protein constructs of PvNV and MrNV are listed in Table 1.

**Table 1:** Various capsid protein constructs of PvNV and MrNV

Strain	Encoding region	Expression vector	*N*/*C*-termini fusion tags
PvNV	PvNV capsid protein (1−368)	SUMO	None
	PvNVPd (250−368)		
	ΔN-ARM-PvNVSd (38−250)		
	ΔN-ARM-PvNV (45−368)		
MrNV	MrNVPd (246−371)		
PvNV	PvNV capsid protein (1−368)	pET21	_1_MASMTGGQQMGRGSEF_16 385_LEHHHHHH_392_

**Fig. 1:**
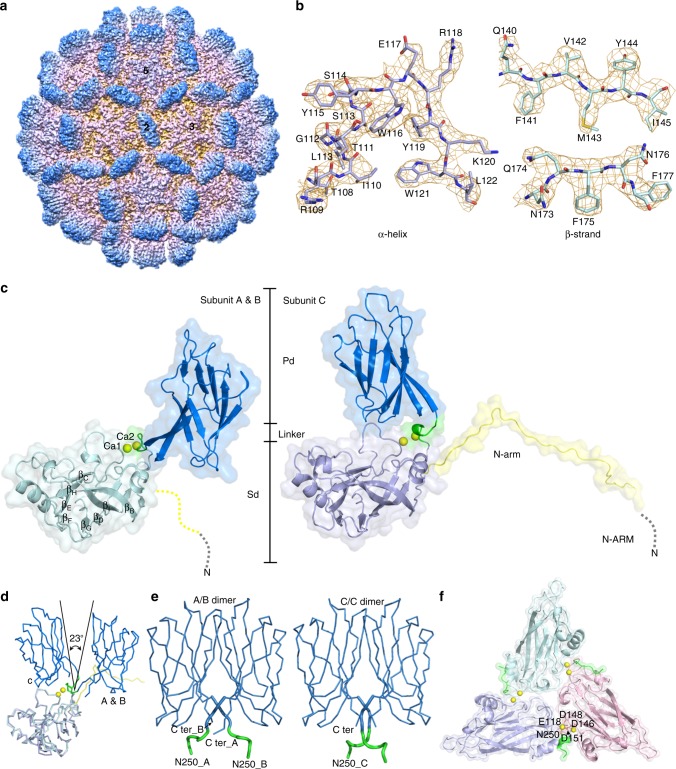
Cryo-EM asymmetric reconstruction of the *T* = 3 PvNV-LP at 3.5 Å resolution. **a** Front view of the cryo-EM map along an I2 symmetry axis with two, three, and five-fold axes as indicated. The capsid surfaces are five-colored according to their distances from the particle center (red: short distance; blue: long distance). **b** Segmented cryo-EM densities (orange mesh) with the corresponding atomic structures (sticks) of the typical α-helix and β-strands, respectively. **c** Ribbon diagrams and molecular surfaces of subunits A and B, and C with bound Ca^2+^ ions (yellow spheres), comprising several domains and regions, including N-ARM (gray), N-arm (yellow), the S-domain (light green and purple), the linker (green) and the P-domain (blue), respectively. **d** The different orientations of the subunits A and B, and C are color-coded as in **c**. **e** The homo-dimeric P-domains and linkers from the A/B and C/C dimers are color-coded as in **c**. Asn250 and the *C*-termini are indicated. **f** The S-domains of the three subunits per iASU. Six Ca^2+^ ions (yellow spheres) are incorporated at the interfaces of neighboring subunits A (light green), B (pink), and C (purple), where Glu118, Asp146, Asp148, Asp151, and Asn250 are involved in the Ca^2+^ binding

The structure of a virion-sized *T* = 3 PvNV-LP was determined by cryo-EM at 3.5 Å resolution (Table 2) (Supplementary Figs. 2 and 3a, b). The cryo-EM map clearly shows *T* = 3 icosahedral symmetries with 90 spikes, composed of 180 copies of the 37-kDa full-length capsid proteins in total, distributed along the icosahedral 2-fold (I2) axes and the quasi 2-fold (Q2) axes on the surface (Fig. 1a). The bulky side chains of α-helices and β-strands in the *T* = 3 PvNV-LP are clearly resolved (Fig. 1b). The crystal structures of the *T* = 1 ΔN-ARM-PvNVSd and PvNVPd, determined individually as described later, were well fitted into the cryo-EM map without major adjustments.

**Table 2:** Statistics of data collection and processing

	*T* = 3 PvNV-LP (EMD-9576) (PDB 6AB6)	*T* = 1 PvNV-LP (EMD-6999) (PDB 6AB5)
*Data collection and processing*		
Magnification	59,000×	
Voltage (kV)	300	
Electron exposure (e–/Å^2^)	40	
Defocus range (μm)	−1.25 to −2.75	
Pixel size (Å)	1.12	
Symmetry imposed	Icosahedral sym.	Icosahedral sym.
Initial model used (ID)	EMD-3655	PDB 2ZZQ
Applied low-pass filter	60	40
Initial particle images (no.)	42,751	476,834
Final particle images (no.)	8596	70,543
Map resolution (Å)	3.5	3.7
FSC threshold	0.143	0.143
*Refinement*		
Resolution (Å)	3.5	3.7
Map sharpening *B* factor (Å^2^)	−195	−273
Model composition		
Nonhydrogen atoms	–	–
Protein residues	–	489
Ligands	–	–
*B* factors (Å^2^)		
Protein	70.36	61.55
Ligand	96.20	–
R.m.s. deviations		
Bond lengths (Å)	0.0077	0.01
Bond angles (°)	1.20	1.18
Validation		
MolProbity score	1.61	1.61
Clashscore	4.69	4.51
Poor rotamers (%)	0.48	0.37
Ramachandran plot		
Favored (%)	94.7	94.4
Allowed (%)	5.3	5.6
Disallowed (%)	0.0	0.0

The icosahedral asymmetric unit (iASU) within the *T* = 3 PvNV-LP contains three capsid proteins occupying inequivalent sites with somewhat different conformations, which will be henceforth referred to as A–C subunits. The cryo-EM map allowed modeling of residues 64−368 for subunits A and B, and residues 31−368 for subunit C (Fig. 1c). In *T* = 3 PvNV and MrNV, these subunits contribute to 2 kinds of surface protrusions: 30 dimeric spikes along the I2 axes and 60 dimeric spikes along the Q2 axes, respectively. The dimeric C/C and A/B capsomeres comprise I2 and Q2 dimeric spikes, respectively. Of the three subunits in the iASU, the P-domains from the A/B and C/C capsomeres form tight dimeric spikes on the surface from two different iASUs. Thus, the *T* = 3 shell exists as slightly different dimeric capsomeres consisting of an asymmetric A/B and a symmetric C/C dimers with the bent and flat conformations, respectively. In the *T* = 3 PvNV-LP, the ordered linkers from the C/C dimer exhibit a parallel structure at the opposite side, whereas the linkers from the A/B dimer adopt another new structural feature with a cross conformation. One linker of the A/B dimer exhibits an open form with an angle of deviation ~23° from the opposite subunit along the Q2 axis compared with the close form of the C/C dimer (Fig. 1d, e).

### The structure of PvNV capsid protein

The overall structure of the PvNV capsid protein comprises four regions, including the *N*-terminal arm (N-arm) (residues 31−63), the S-domain (64−249), the linker (250−255), and the P-domain (256−368), existing in two distinct structural forms for the A/B and C subunits (Fig. 1c). The *N*-terminal segment of each subunit, which contains the positively charged arginine-rich motif (N-ARM) with _22_RRVRGGRVSRRR_33_, protrudes into the capsid inner cavity in a disordered conformation. The linker connecting the S-domain to the P-domain is located beneath the surface spike.

The S-domain of *T* = 3 PvNV-LP adopts a typical jelly-roll β-barrel fold as a canonical structural feature (Fig. 1c). The eight β-strands, designated with letters B to I, form an antiparallel-barrel structure with two pairs of four-stranded antiparallel sheets, called BIDG and CHEF, which are connected with several loops and four helices of various lengths, respectively (Fig. 1c). Despite the conserved central β-sheet structure, the PvNV S-domain shares low-sequence identity with other viral capsid proteins. A search with the *DALI* program reveals that the PvNV S-domain shares some structural homology with S-domains of several viral capsid proteins, such as the orsay virus (PDB 4NWV; *z*-score 18.8)^16^, grouper nervous necrosis virus (4RFT; 18.0)^13^ and carnation mottle virus (1OPO; 17.9)^17^.

### Ca^2+^ ions of the PvNV S-domain

Remarkably, one iASU of the *T* = 3 PvNV-LP contains three sets of two Ca^2+^ ions, located at the interfaces between subunit pairs, which are coordinated with side chains of Asp146, Asp148 and Asp151 that form a _146_**D**x**D**xx**D**_151_ motif at the E–F loop, Glu118 at the C–D loop, and Asn250 beside the linker from the neighboring subunit (Fig. 1f). This organization is similar to those of betanodavirus (**D**xx**D**xD)^13^ and several RNA plant viruses, such as tombusvirus (**D**x**D**xx**D**)^18^ and sesbania mosaic virus (SeMV) (**D**xx**D**)^19^. Divalent metal ions, such as Ca^2+^, play critical roles in the formation, stability, and infectivity of a capsid^20^.

### Structural characterization of the *N*-terminus of PvNV capsid protein

**Fig. 2:**
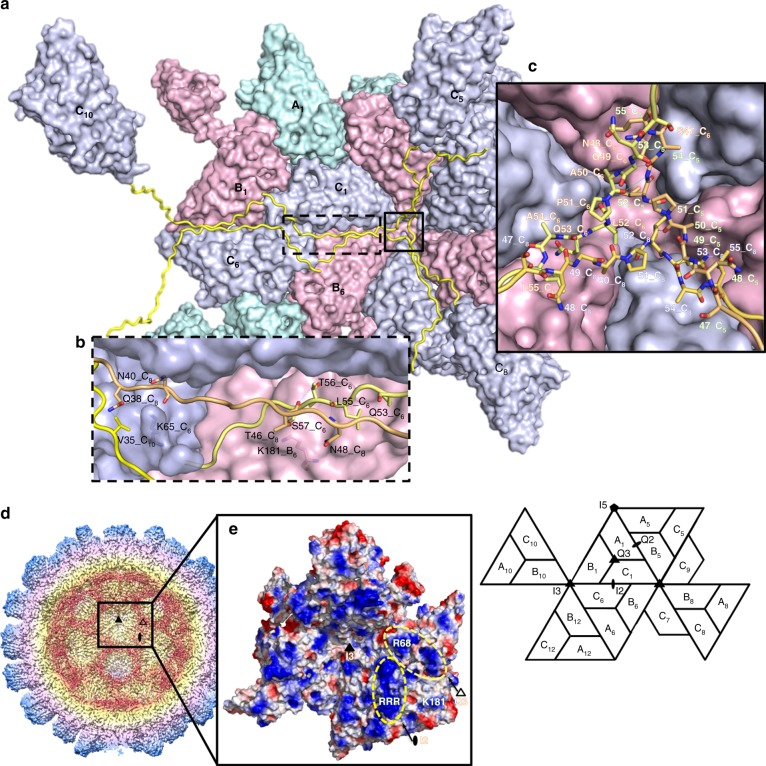
Structural organizations of the N-arm and the β-annulus. **a** A surface presentation of the C/C dimer along the I2 axis and the hexameric capsomeres along the I3 axis as viewed from the inner surface. Subunits A–C are colored in light green, pink, and purple, respectively. N-arms are colored in yellow. **b** The three N-arms from three subunits C_6_, C_8_, and C_10_ along the I2 groove. The key residues from three subunits C are involved in the tetrameric organization of the ordered N-arms contributed by hydrogen bonds. **c** Residues 47–55 of three different subunits C form a β-annulus structure as an enlarged central section. The hydrogen bonds are shown with blue dotted lines. **d** A cutaway view of the *T* = 3 PvNV-LP. A half-sectional view of the *T* = 3 PvNV-LP reveals the interaction of the S-domains with the extra average density (red) around inner surface. **e** The electrostatic potential surface of the S-domains. The locations (yellow dotted lines) near the I2 and Q3 axes represent the positively charged residues at the inner surface. The positively charged residues including _31_RRR_33_, Arg68, and Lys181 are shown in cyan

The semiflexible basic N-ARM (residues 1–30) embedded within the inner cavity might be involved in viral nucleic-acid encapsidation. It has been proposed that capsid proteins use the highly basic N-ARM to control both the length and conformation of the genome with nonspecific electrostatic interactions in ssRNA viruses^21^. The particularly long N-arm (residues 31–63) is clearly resolved along the I2 interface for subunit C; this ordered N-arm extends to the icosahedral 3-fold (I3) axis and exhibits long-range antiparallel like segment–loop interactions (Fig. 2a, b).

The four extended ordered N-arms from C_1_/C_6_ and C_8_/C_10_ occupy the groove of the inner surface along one I2 interface (Fig. 2a, b). These four N-arms exhibit distinct symmetric tertiary structures within the inner surface of the C_1_/C_6_ dimer, which are the first discovered in the capsid structures. Surprisingly, two *N*-terminal ends of the N-arms from C_8_ and C_10_, presumably together with their corresponding N-ARM, touch the inner surface of the C_1_/C_6_ dimer and hang slightly beneath subunits B_1_ and B_6_ near the corresponding quasi 3-fold (Q3) axes, respectively. The buried N-arms of subunits C_6_, C_8_, and C_10_ interact with two positively charged side-chains of Lys65 and Lys181 from two subunits C_6_ and B_6_ through hydrogen bonding (Fig. 2a, b). Furthermore, residues 47–55 of the N-arms from subunits C_5_, C_6_, and C_8_ are engaged with hydrogen bonds to form a β-annulus structure around the I3 axis (Fig. 2a, c). In this β-annulus structure of the *T* = 3 PvNV-LP, these hydrogen-binding interactions are established potentially with the structurally equivalent carbonyl group of Ala50 and side-chain carbonyl group of Leu52, respectively. The nearby Pro51 contributing to this region is structurally conserved and equivalent to Pro38 in betanodavirus^13^, Pro35 in rice yellow mottle virus (RyMV)^22^, and Pro53 in SeMV^19^. With the same location of the I3 axis, the β-annulus structure of PvNV is similar to those of the betanodavirus and RyMV, but differs from that of the SeMV, comprising three N-arms from subunits C_1_, C_7_, and C_9_.

Interestingly, along the I2 and Q3 axes of the *T* = 3 PvNV-LP, some extra density from the icosahedral symmetric reconstruction cryo-EM map was observed. These extra average densities, protruding from the inner surface of the shell, might reflect endogenous RNA molecules interacting directly with partially disordered N-ARM (Fig. 2d). Figure 2e shows an electrostatic potential distribution on the inner surface of the *T* = 3 PvNV-LP. A close inspection reveals that three positively charged residues _31_RRR_33_, located at the tip of the long N-arm near the I2 axis, might help to anchor the coat proteins to the negatively charged nucleic acid. Furthermore, Arg68 and Lys181 of the S-domain, exposed to the inner surface around the Q3 and icosahedral 5-fold (I5) axes, might facilitate further participation of the encapsidated RNA to participate in the *T* = 3 quaternary organization (Fig. 2e).

### Overall structures of the PvNV and MrNV P-domains

In the cryo-EM structure of the *T* = 3 PvNV-LP, the morphology of the 90 dimeric surface spikes can be readily assigned and shown to differ from those of the two major genera of the family *Nodaviridae* with trimeric protrusions (Supplementary Fig. 4a). An insufficient electron density of the dimeric spike initially limited a complete characterization of the P-domain at atomic resolution. Accordingly, we prepared both the P-domains of PvNV and MrNV, the truncated PvNVPd (residues 250–368) and MrNVPd (245–371) (Table 1) (Supplementary Fig. 4b). Both PvNVPd and MrNVPd (without the SUMO-tag) were eluted as dimers by size-exclusion chromatography. Sodium dodecyl sulfate polyacrylamide gel electrophoresis (SDS-PAGE) analysis of both proteins yielded molecular masses ~13 and ~26 kDa for monomers and dimers, respectively.

The PvNVPd forms dimers in two crystal forms with the asymmetric unit containing one dimer (space group *P*2_1_) and two dimers (*P*2_1_2_1_2_1_). The PvNVPd structure (*P*2_1_) were first determined using Se-Met-derivatized crystals with single-wavelength anomalous diffraction (SAD). All structures of the PvNVPd were refined to resolutions 1.17–1.22 Å (Table 3). The structures of PvNVPd from two crystal forms are similar with a root mean square deviation (RMSD) < 0.3 Å; and residues 250–368 were resolved in both structures.

As for the MrNvPd, three crystal forms (*P*3_2_21, *C*222_1_, and *P*4_3_32) were determined. The structure of MrNVPd (*P*3_2_21) was determined by Se-SAD, independently from PvNVPd, and all structures were refined to 1.39–2.3 Å resolutions (Table 3). The overall conformations and dimer interfaces in three crystal structures of MrNVPd are similar with a RMSD < 0.3 Å. Residues 249–371 were resolved in the structure at resolution 1.39 Å.

**Table 3:** Data collection and refinement statistics

	PvNVPd *P*2_1__Peak	PvNVPd *P*2_1_ (5YKZ)	PvNVPd *P*2_1_2_1_2_1_ (5YL0)	MrNVPd *P*3_2_21_Peak	MrNVPd *P*3_2_21 (5YKU)	MrNVPd *C*222_1_ (5YKV)	MrNVPd *P*4_3_32 (5YKX)	*T* = 1 ΔN-ARM-PvNVSd SVPs (5YL1)
*Data collection*								
Beamline^a^	TLS 15A1	TLS 15A1	BL44XU	TLS 13C1	TLS 13C1	BL44XU	BL44XU	TPS 05A
Wavelength (Å)	0.979	1.000	0.900	0.976	0.976	0.900	0.900	1.000
Temperature (K)	110	110	100	110	110	100	100	110
Space group	*P*2_1_	*P*2_1_	*P*2_1_2_1_2_1_	*P*3_2_21	*P*3_2_21	*C*222_1_	*P*4_3_32	*P*2_1_2_1_2_1_
*Cell dimensions*								
*a*, *b*, *c* (Å)	42.72, 53.14, 46.38	42.60, 53.19, 46.07	43.84, 69.74, 146.61	72.87, 72.87, 47.04	72.87, 72.87, 47.04	125.77, 147.28, 175.92	134.18, 134.18, 134.18	196.98, 200.32, 419.29
*α*, *β*, *γ* (°)	90.00, 109.52, 90.00	90.00, 109.38, 90.00	90.00, 90.00, 90.00	90.00, 90.00, 120.00	90.00, 90.00, 120.00	90.00, 90.00, 90.00	90.00, 90.00, 90.00	90.00, 90.00, 90.00
Resolution (Å)^b^	30–1.28 (1.33–1.28)	30–1.17 (1.21–1.17)	30–1.22 (1.26–1.22)	30–1.39 (1.44–1.39)	30–1.39 (1.44–1.39)	30–2.30 (2.38–2.30)	30–2.00 (2.07–2.00)	30–3.12 (3.23–3.12)
*R*_sym_ (%)^b^	3.6 (16.7)	7.7 (41.0)	11.2 (43.8)	7.0 (33.7)	7.0 (33.7)	14.2 (63.0)	11.2 (83.0)	21.8 (86.0)
*I*/σ_*I*_^b^	3.6 (16.7)	7.7 (41.0)	11.2 (43.8)	7.0 (33.7)	7.0 (33.7)	14.2 (63.0)	20.3 (19.0)	5.0 (5.0)
Completeness (%)^b^	98.2 (96.6)	99.1 (92.4)	86.9 (86.9)	99.2 (98.4)	99.2 (98.4)	99.3 (97.6)	99.9 (99.8)	99.7 (99.8)
Redundancy^b^	8.3 (8.3)	4.6 (4.3)	4.5 (4.8)	9.6 (9.6)	9.6 (9.6)	5.3 (4.9)	20.3 (19.0)	5.0 (5.0)
*Refinement*								
Resolution (Å)		30–1.17	30–1.22		30–1.39	30–2.30	30–2.00	30–3.12
No. of reflections		58,534	110,940		27,637	66,484	27,072	274,292
*R*_work_/*R*_free_		15.2/16.9	17.5/22.0		14.2/15.1	16.0/19.8	15.6/19.5	21.9/22.1
*No. of atoms*								
Protein		1681	3321		958	7748	1880	1454
Ligand		–	–		–	–	15	–
SO_4_		–	–		–	–	5	–
Metal		–	–		3(Zn)	–	3(Cd)	2 (Ca)
Water		414	1477		275	736	308	–
*B-factors*								
Protein		16.43	14.35		12.25	29.90	27.26	64.58
Ligand		–	–		–	–	80.26	–
SO_4_		–	–		–	–	48.65	–
Metal		–	–		10.97	–	25.54	73.41
Water		30.03	36.67		27.90	38.87	52.11	–
*R.m.s. deviations*								
Bond lengths (Å)		0.027	0.027		0.028	0.011	0.022	0.011
Bond angles (°)		2.117	2.137		2.368	1.447	2.221	1.696

^a^One crystal for each structure was used

^b^Values in parentheses are for highest-resolution shell

**Fig. 3:**
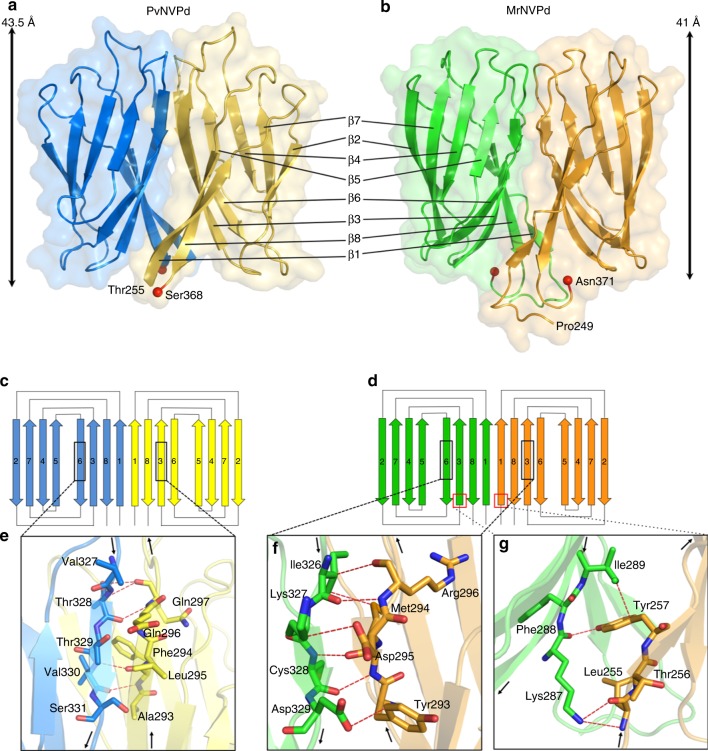
Topology and structural organization of the PvNVPd and MrNVPd. **a**, **b** Ribbon diagrams and surface presentations of the PvNVPd and MrNVPd are shown in similar orientations. The two *C-*termini are shown as red spheres. Each monomer of PvNVPd and MrNVPd is indicated in blue, yellow, green, and orange, respectively. **c**, **d** Schematic diagrams of the secondary structures in the PvNVPd and MrNVPd, respectively. Each monomer of PvNVPd and MrNVPd is indicated with colors as in **a** and **b**. **e**–**g** The hydrogen bonds, which stabilize the dimer, from specific β-strands β1, β3 and β6 with the key residues (sticks) at the interface are shown in red

**Fig. 4:**
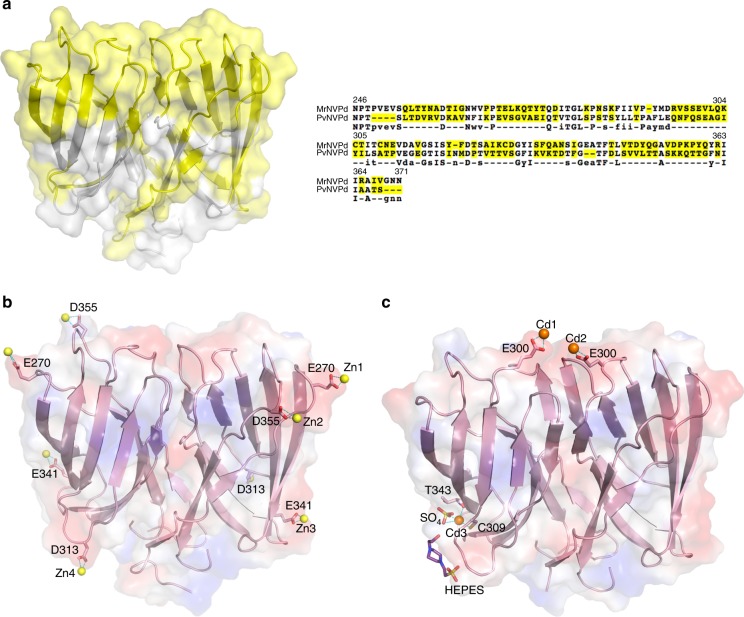
Insights on the surface of one dimeric MrNVPd. **a** Sequence-alignment variables between MrNVPd and PvNVPd mapped onto the surface of the P-domain. The hypervariable regions (yellow) from MrNVPd and PvNVPd are presented on the molecular surface and strictly variants residues are colored in yellow as well. **b** The molecular electrostatic potential surface of the dimeric MrNVPd with the Zn^2+^ ions. A side view of the dimeric MrNVPd is colored in red and blue for negatively and positively charged regions, and the Zn^2+^ ions are shown in yellow spheres. **c** The side view of the MrNVPd with the Cd^2+^ ions (orange spheres). The ligands of SO_4_ and HEPES are colored in yellow and blue, respectively

Consistent with the cryo-EM structure and size-exclusion chromatography, both PvNVPd and MrNVPd form a dimeric straight rod with dimensions of length ~40 Å and width ~22 Å in crystal packing (Fig. 3a, b). The secondary-structure elements are shown in Fig. 3c, d. The structural fold of the PvNVPd consists of eight β-strands, differing from that of any other known viral capsid protein. The semi-flexible *N*-terminal linker (residues 250–255) located beneath the P-domain must be disordered as there is absence of electron density (Fig. 3a). The P-domain monomer folds into the core jelly-roll topology, forming a β-sandwich face-to-face with two pairs of antiparallel β-sheet. Four highly exposed turn insertions β1/β2, β3/β4, β5/β6, and β7/β8 appear on the top regions of the PvNVPd and might play an important role in the determination of the antigenicity. The MrNVPd has a structural fold consisting of eight long stretches of antiparallel β-strands similar to the PvNVPd, but shares a low sequence homology with PvNVPd (Fig. 4a and Supplementary Fig. 5). The *C*-termini of PvNVPd and MrNVPd are both located near the linker at the interface space between the S- and P-domains. Superimposition of PvNVPd and MrNVPd monomers, comprising 113 and 122 Cα positions, respectively, yielded a RMSD of 1.6 Å. Furthermore, the structural alignment of PvNVPd and MrNVPd with all structures using *DALI* reveals a low structural similarity (*z*-score < 6.5), indicating that the P-domain exhibits a new structure.

### Interface interactions in the PvNVPd and MrNVPd dimers

In the dimeric structures of PvNVPd and MrNVPd, the domain–domain contacts at the interface are 2-fold symmetrically formed with a hydrogen-bond network between the main chains of the antiparallel β-strands, β3 and β6 (Fig. 3c, e). Several residues are involved in these interactions, including Ala293–Gln297 and Val327–Ser331 for PvNVPd and Tyr293–Arg296 and Ile326–Asp329 for MrNVPd, respectively (Fig. 3d, f). In addition, the MrNVPd dimer is stabilized with hydrogen bonds between Leu255 and Tyr257 of the β1 strand and Lys287 and Ile289 of the β3 strand (Fig. 3g).

To investigate further whether the two β-strands β3 and β6 play important roles in dimerization, we generated two double-mutants (Y293A/D295A and K327A/D329A) of MrNVPd. As expected, the size-exclusion chromatography and crystallography show that both double-mutants could still form dimeric structures, indicating that hydrogen-bonding interactions between the main chains of these two major β-strands are critical in the dimerization process.

Previous studies show that two face-to-face β-sheets composed of five β-strands are found along the 2-fold axes of the protrusion to provide the strong interaction to drive the dimeric association in the tomato bushy stunt virus (TBSV)^23^, whereas dimerization of the P2-domains of HEV is mediated by three β-strands from the central β-barrel and one *C*-terminal loop through the hydrophobic interaction at the 2-fold interface^24,25^ (Supplementary Fig. 6). Obviously, PvNVPd and MrNVPd show no notable sequence similarity with TBSV and HEV, and exhibit a distinctive feature that the dimer is formed through hydrogen-bonding interactions located at a flat interface.

### Divalent metal-ion binding in the MrNVPd dimer

An analysis of the electrostatic potential distribution of the MrNVPd dimer shows a mostly negatively charged surface. First, the high-resolution crystal structure (*P*3_2_21) allows us to locate clearly the metal ions (Fig. 4b). The _311_Ex**D**_313_ motif on turn β4/β5 interacts with one Zn^2+^ ion through direct electrostatic interactions. Glu270 on turn β1/β2 and Asp355 on turn β7/β8 also coordinate with Zn^2+^ ions. The distances between the Zn^2+^ ions and the side chains of Glu270, Glu341 and Asp355 from three separate asymmetric units are ~2.4 Å, which is in a reasonable range for a Zn^2+^/ligand coordination^26^. In addition, these three key residues are involved in another Zn^2+^-binding site located at the intermolecular interface of two MrNVPd dimers.

Second, the structure of MrNVPd (*P*4_3_32) reveals an additional metal-binding site involved coordinating with both oxygen atoms from the side-chain carboxyl groups of two Glu300 residues. On the basis of the environment of the crystal condition, it is likely to be a divalent metal, such as cadmium (Cd^2+^) (Fig. 4c). These two glutamic-acid residues from one MrNVPd dimer are conserved in PvNVPd (Glu302). The side chains of the two Glu300 residues on turn β3/β4 of the MrNVPd dimer alter the conformation upon coordinating the Cd^2+^ ions. The distances for the Cd^2+^-ligand oxygens from the two Glu300 residues are ~2.4 Å (Fig. 4c). Finally, the initial difference map shows one outstanding electron density peak near residues Cys309 and Thr343, which is interpreted as one Cd^2+^ ion. This Cd^2+^ ion is coordinated to the sulfur atoms from the side chain of Cys309 residue, one sulfate molecule from the crystallization buffer, and a backbone carbonyl oxygen atom from the main chain carboxyl groups from residues Thr343. This second Cd^2+^-binding site stabilizes the MrNVPd by bridging the residues from the flexible turn β4/β5 to the β-strand β7 of the jelly-roll structure.

### Organization of the P-domain dimers on *T* = 3 shrimp nodavirus

**Fig. 5:**
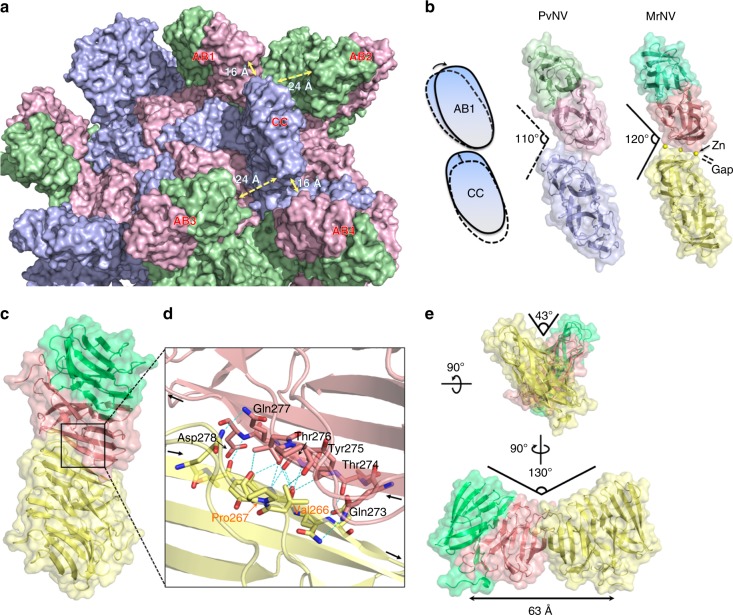
Intermolecular conformations of the dimeric P-domains. **a** The outer surface presentation of dimeric P-domains. The subunits A–C are colored in light green, pink, and purple, respectively. **b** The top views of the dimeric P-domains corresponding to the subunit-A/B and C/C dimers of the *T* = 3 PvNV-LP. The cartoon presentation of dimeric P-domains movement between A/B dimer and C/C dimer. The two PvNVPd dimers are engaged in the parallel conformation and are colored as subunits A–C in green, pink, and purple, respectively; the two MrNVPd dimers are engaged in the parallel conformation and the subunits A–C are colored in light green, salmon, and light yellow, respectively. The Zn^2+^ ions are located at the interface, resulting in a gap between two MrNVPd dimers. **c** A top view of the X-shaped conformation of the dimer–dimer MrNVPd. The two MrNVPd dimers are shown as blue and salmon, respectively. **d** The 2-fold dimerization interface of two MrNVPd dimers. The blue dotted lines indicate intermolecular hydrogen bonds between two MrNVPd dimers as colored in **c**. **e** The crossing angles of the two MrNVPd dimers. These two angles are stabilized by intermolecular hydrogen bonds, and are presented from two side views

An inspection of the surface spikes of *T* = 3 PvNV-LP reveals great differences of the gap distances between the two nearby dimeric spikes formed by the A/B and C/C dimers, with ~24 Å between subunits A and C and ~16 Å between subunits B and C, due to the special orientation of the dimeric P-domains in the C/C dimer (Fig. 5a). A previous low-resolution cryo-EM map of the *T* = 3 MrNV-LP showed that the A/B and C/C dimeric P-domains are orientated 105° around the I3 axes and the two leaning A/B P-domain dimers form contacts with one raised C/C P-domain dimer, resulting in a larger blade-like superstructure (A/B–C/C–A/B) around the I3 axes^15^. A comparison of our *T* = 3 PvNV-LP structure and the low-resolution MrNV-LP map also shows a major difference in the stoichiometry of the surface P-domain spikes (Supplementary Fig. 7a, b).

Our high-resolution crystal structures of PvNVPd and MrNVPd reveal distinct temperature *B*-factors of the P-domains and linkers, indicating that the dimeric P-domains are rigid and stabilized by the intermolecular network of hydrogen bonds, whereas the linkers are flexible, which might influence both the positions and organizations of the 90 dimeric spikes on the *T* = 3 PvNV and MrNV virions. We consequently present two distinct putative dimer–dimer conformations to mimic the multiple organizations of the dimeric spikes: a parallel model and a closed contact as a X-shaped model structure, in which the two antiparallel β-strands associated with each other form a hydrogen-bond network in one PvNVPd and two MrNVPd crystal structures that are described as follows.

First, the crystal packing of PvNVPd (*P*2_1_2_1_2_1_) reflects the intermolecular organization of the two P-domain dimers with a bending angle of ~110°, suggesting that the existence of this putative conformation in the *T* = 3 PvNV virions arises from the simultaneous rotation of A/B dimer and the translation of C/C dimer (Fig. 5b). In the interface of this parallel model, the three turns β1/β2, β6/β7, and β7/β8 from one PvNVPd monomer are in close proximity to those of one symmetry molecule in the absence of metal ions. Similar to PvNVPd, the symmetry of MrNVPd dimers (*P*3_2_21) also generates a dimer–dimer conformation, but with additional metal ions coordinated in the dimer–dimer interface and with a slightly altered bending angle of ~120° (Fig. 5b). Three key residues Glu270, Glu341, and Asp355 reveal a negatively charged interface and work together with three Zn^2+^ ions through hydrogen bonds connecting three turns β1/β2, β6/β7, and β7/β8, respectively (Fig. 4b). Examination of the intermolecular MrNVPd dimer–dimer interaction reveals that Glu270, Glu341, and Asp355 donate major hydrogen bonds to stabilize this conformation (with the exception of the _311_Ex**D**_313_ motif), suggesting that the negatively charged Asp313 on the MrNVPd monomer might exist as a potential metal-binding site without participating in the MrNVPd dimer–dimer structure.

Second, two MrNVPd dimers (*C*222_1_) in an asymmetric unit form an X-shaped model with the antiparallel β-strand interaction through an extensive network of hydrogen bonds between residues from two β-strands β2 (Gln273–Asp278) and two turns β1/β2 (Val266–Pro267) (Fig. 5c–e). The two dimers in the X-shaped model are orientated with a tilt angle of 130° and with separation of ~63 Å between the two linker terminals. Notably, the sequence of the interaction region (Gln273–Asp278) of MrNVPd is distinct from that of PvNVPd, and two types (*P*2_1_ and *P*2_1_2_1_2_1_) of PVNVPd structures do not exhibit the X-shaped conformation. It appears that the X-shaped dimeric MrNVPd organization reveals no homology compared with intermolecular P-domain conformations of other viral capsids.

### Particle polymorphism of *T* = 3 and *T* = 1 capsids in PvNV-LP

**Fig. 6:**
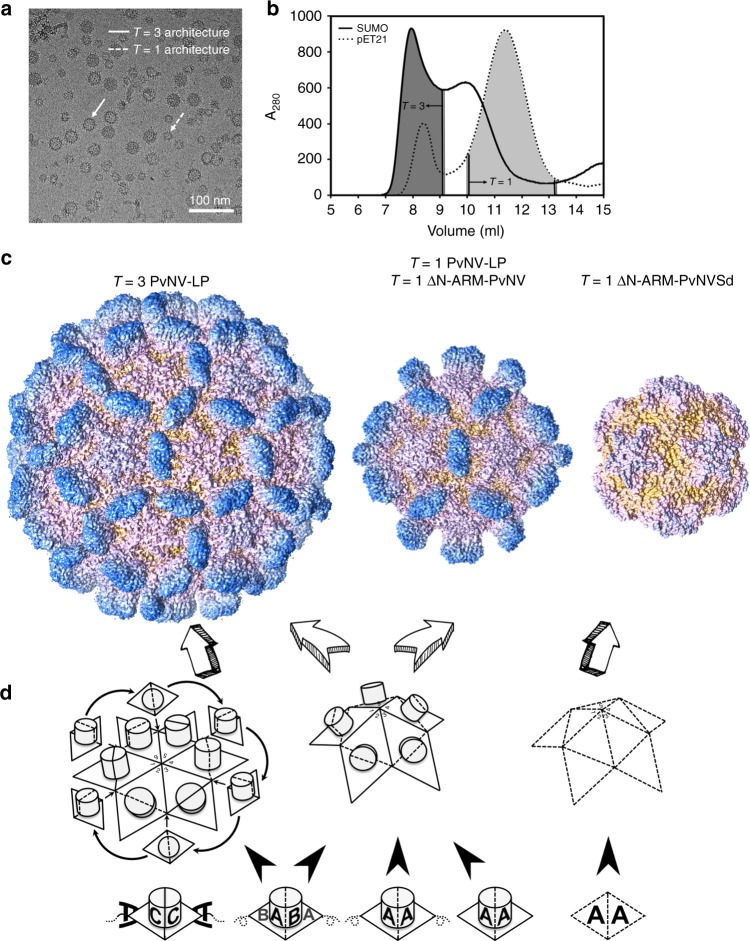
Particle polymorphism and self-assembly mechanisms of shrimp nodavirus. **a** Cryo-EM image of the PvNV-LP. The diameters of the PvNV-LPs are near 35 and 24 nm, corresponding to the *T* = 3 and *T* = 1 particles. *T* = 3 and *T* = 1 capsids are indicated by white solid and dotted arrows, respectively. Scale bar = 100 nm. **b** The elution profiles of the PvNV-LP and pET21-PvNV-LP in the size-exclusion chromatography. The various fractions were collected and their absorbance at 280 nm were measured. **c** Cryo-EM, X-ray crystal and EM structures of the *T* = 3 and *T* = 1 PvNV-LPs, *T* = 1 ΔN-ARM-PvNV SVP and *T* = 1 ΔN-ARM-PvNVSd SVP. The *T* = 3 PvNV-LP surface and two *T* = 1 SVPs surfaces are rainbow-colored according to their distance from the spike to shell. **d** The diagram shows the putative self-assembly process of the *T* = 3 and *T* = 1 shrimp nodavirus capsids. The basic unit of capsid assembly is the dimeric capsomeres (diamond with black solid and dotted lines). The N-ARM deletion and the *N*-terminal fusion tag both might guide the assembly of the *T* = 1 capsid

After SUMO-tag cleavage, the PvNV-LPs self-assemble in vitro with no fusion segment. Visualization by cryo-EM showed that these capsids exist in multiple conformations, including mostly virion-sized *T* = 3 particles of diameter ~35 nm and a minor proportion of smaller-sized *T* = 1 particles of ~24 nm (Fig. 6a).

The structure of a smaller-sized *T* = 1 PvNV-LP particle was determined by cryo-EM at 3.7 Å resolution (Table 2) (Fig. 6c and Supplementary Figs. 2, 3a, b). The subunit A of the *T* = 3 PvNV-LP was used to assist in the building of a complete model for the *T* = 1 PvNV-LP. The final atomic model of a capsid protein in the *T* = 1 PvNV-LP includes residues 64–368 without the mostly structurally disordered *N*-terminal region (residues 1–63), and comprises a canonical viral jelly-roll S-domain with two four-stranded antiparallel β-sheets (BIDG and CHEF), a linker and a P-domain. Totally, 60 PvNV capsid proteins were structurally equivalent with the I2, I3, and I5 axes. A structural comparison of one subunit of *T* = 1 PvNV-LP and the subunit A of *T* = 3 PvNV-LP reveals RMSD < 0.2 Å. The 30 spikes on the outer surface are also readily discernible. They might be mainly involved in the homo-dimeric capsomere interactions, resulting in the A/A dimer with a bent conformation with angle ~144°, similar to the A/B dimer of *T* = 3 PvNV-LP (Supplementary Fig. 7c). Although there is no extra density at the interface between two neighboring subunits of the *T* = 1 PvNV-LP, the high structural homology of the S-domains in the *T* = 3 and *T* = 1 PvNV-LPs suggests that the Ca^2+^-binding site is conserved.

To clarify further whether or not fusion segments on recombinant PvNV capsid proteins affect the assembly of these multiple conformational particles, we attempted assembly in vitro of the full-length PvNV capsid protein with another expression vector, pET21 (Table 1). In comparison with PvNV-LP with no SUMO fusion segment, we found that the pET21-PvNV-LP from containing the *N*/*C*-termini fusion segments with the 6× His-tag might have the propensity to self-assemble into highly irregular particles of the *T* = 1 architecture, according to EM (Supplementary Fig. 4b, c). According to size-exclusion chromatography, the particle populations of these two PvNV-LPs samples indicate that pET21-PvNV capsid protein generates a heterogeneous population of the viral particles (Fig. 6b).

### N-ARM as a molecular switch for *T* = 1 subviral particles

The N-ARM has been shown to play a role in controlling the particle polymorphism, such as cowpea chlorotic mottle virus^27^, SeMV^19,28^, TBSV^29^, cucumber necrosis virus^30^, hibiscus chlorotic ringspot virus^31^, and GNNV^13^. To examine whether or not the shrimp nodavirus capsid proteins could assemble into distinct particles in the absence of N-ARM, the crystal structure of the truncated PvNVSd (ΔN-ARM-PvNVSd, residues 38–250) was determined at resolution 3.1 Å and showed a *T* = 1 SVP architecture (Table 3) (Fig. 6c). In the final atomic model, the ΔN-ARM-PvNVSd comprising residues 66–250, without the disordered *N*-terminal region (38–65), organizes into the prevalent jelly-roll β-barrel fold with adjacent eight antiparallel β-strands. The *T* = 1 ΔN-ARM-PvNVSd SVP contains 120 Ca^2+^ ions occupying the Ca^2+^-binding sites at the interface of each subunit, which are conserved with the *T* = 3 PvNV-LP.

In support of the conclusion that N-ARM serves as a molecular switch to control particle sizes, the negative-staining EM image of the ΔN-ARM-PvNV SVP (residues 45–368) was also recorded (Table 1). The EM image showed that the ΔN-ARM-PvNV indeed form a *T* = 1 capsid of diameter ~24 nm consistent with *T* = 1 PvNV-LP (Fig. 6c and Supplementary Fig. 4d).

## Discussion

The cryo-EM structure of *T* = 3 PvNV-LP reveals the especially long ordered polypeptide of the N-arm, which makes a specific arrangement through the β-annulus structure with three conserved proline residues (Pro51) from three subunits C_5_, C_6_, and C_8_, resulting in an *N*-terminal extended arch-shaped structure and the flexible N-ARM located around the Q3 axis. The flat contact of the C_1_/C_6_ dimer seems able to create a spacious locus to accommodate the two N-arms (C_1_ and C_6_) and another two N-arms (C_8_ and C_10_), which have never been observed in other known *T* = 3 viruses. MrNV has the similar sequence length of the N-arm and contains the conserved proline residue (Pro48). We thus surmise that the N-arm and β-annulus structures of MrNV are similar to those of the PvNV.

The PvNV genome comprising the 4294 nt-long RNA molecule is supposed to interact with the inner surface of the *T* = 3 capsid according to the specific position of the N-ARM and the distribution of the positively charged residues on the S-domain. Our cryo-EM map of the recombinant *T* = 3 PvNV-LP contain some information about the icosahedral-averaged endogenous RNA structure. Partial electron densities corresponding to the semiflexible residues and nucleotides are observed to be associated with each S-domain near axes I2 and Q3. The locations of these electron densities might indicate that the viral genome interacts with the specific positively charged residues of the N-ARM and S-domain. The *T* = 3 and *T* = 1 PvNV-LPs have the similar structures of the S-domain, linker, and P-domain, but the N-ARM and N-arm of *T* = 1 PvNV-LP are more flexible. In contrast with the *T* = 3 PvNV-LP, *T* = 1 PvNV-LP, with only A/A dimers, has a bent conformation and a smaller inner cavity, and would associate weakly with the RNA nucleotide, rendering free or partial viral genome encapsidation.

The oligomerization of capsid proteins is the first intermediate step during assembly of a viral capsid. The observation of dimeric spikes of relatively rigid P-domains along the 2-fold axes connecting with the semiflexible linker is important for *T* = 3 PvNV-LP and MrNV-LP. The analyses with size-exclusion chromatography and crystal structures show that PvNVPd and MrNVPd form homo-dimers with extensive intermolecular hydrogen bonds, suggesting that the appropriate accommodation of P-domains might play a prominent role in the dimerization of the capsid protein during the initial assembly of *T* = 3 PvNV and MrNV capsids. Our structures of the *T* = 3 PvNV-LP, *T* = 1 PvNV-LP, PvNVPd, and MrNVPd at present suggest a proposed model in which the functional P-domain might be involved in the dimeric capsomere formation during a capsid assembly of the shrimp nodavirus (Fig. 6d).

PvNV and MrNV contain several hypervariable regions of the amino-acid sequence on their P-domains. The high-resolution structures of MrNVPd reveal several divalent metals bound on the surface. PvNVPd and MrNVPd have similar electrostatic potential surface with negatively charged residues, including Glu269, Glu302, _314_ExE_316_, and Asp345 of PvNV and Glu270, Glu300, _311_Ex**D**_313_, and Glu341 of MrNV, despite their low-sequence identity, implying that PvNVPd might potentially bind divalent metal ions. Moreover, we found that several hypervariable regions coincide with the top regions of the P-domain (Fig. 4a). This observation might well correlate with an evolutionary divergence, resulting in distinct strains of the shrimp nodavirus with host specificities. The functional P-domains on virions are essential for virus–host interaction and the host susceptibility^32–34^. The successful specific host adaptations among varied subtypes in one species have been shown to require particular amino acids essential to the receptor-binding specificity^35^.

The dimeric P-domains are arranged with 30 spikes along the I2 axes and 60 spikes along the Q2 axes on one *T* = 3 PvNV. Our *T* = 3 PvNV-LP and previous MrNV-LP exhibit different rotation and translation of the dimeric P-domains. Such movements of the P-domains have been observed also in slow bee paralysis virus and deformed wing virus under conditions of high salt or low pH^36,37^. To explain the arrangement of surface dimeric P-domains, our crystal structures of the PvNVPd and MrNVPd impose two possibilities of spatial organizations: a parallel conformation or an X-shaped conformation through rolling and tilting over the *T* = 3 virion surface. Although the X-shaped model is observed only in the MrNVPd structure, we speculate that this organization might both exist also on the *T* = 3 MrNV and PvNV virions under various environment even high salt or low pH. Accordingly, the dimeric spikes are expected to be subjected to multiple organizations by means of the flexible and extent linker regions underneath the cementing dimeric P-domains.

Our recombinant PvNV capsid proteins assembled in vitro into three PvNV-LP categories, i.e., *T* = 3 PvNV-LP, *T* = 1 PvNV-LP, *T* = 1 ΔN-ARM-PvNV SVP and *T* = 1 ΔN-ARM-PvNVSd SVP, with corresponding diameters 350, 240, and 190 Å, respectively (Fig. 6c). The full-length recombinant PvNV capsid protein assembled virtually exclusively into 80% *T* = 3 particles and 20% *T* = 1 capsids. In contrast, the pET21-PvNV-LP containing *N*/*C*-termini fusion segments show a population ratio 1:1 of *T* = 3 and *T* = 1 particles (Fig. 6a, b and Supplementary Fig. 4c). The presence of additional fusion segment of pET21-PvNV-LP likely increases the freedom, resulting in the particle polymorphism. This result is likewise reminiscent of varied orientations and dihedral angles, the so-called freedom and restraint, of dimeric capsomeres that could result in varied particle sizes, depending on the inherent flexibility on the *N*- or *C*-termini of viral capsid proteins^27,38^.

The N-ARM of PvNV and MrNV capsid proteins are highly positively charged with several arginine and lysine residues. The positively charged residues of MrNV capsid protein have been shown to play an important role in the nuclear localization in Sf9 cells^39^. In addition, our two PvNV capsid protein deletion mutants both assemble into *T* = 1 SVPs, suggesting that a deletion of the N-ARM seems sufficient to produce a *T* = 1 capsid. Furthermore, the association between a C/C dimer and a nucleotide might be implicated in a size switch between *T* = 1 and *T* = 3 particles. We thus propose a mechanistic model framed around a nucleotide-mediated C/C dimer and a A/B or A/A dimer with the parallel and cross conformations of linkers, respectively, to explain the dynamic assemblies of the *T* = 1 and *T* = 3 PvNV-LPs.

The structures of the two *T* = 3 shrimp nodaviruses differ from those of alpha and betanodaviruses of family *Nodaviridae*, and enable the construction of a structure-based phylogenetic tree comprising other viruses of families *Nodaviridae*, *Sebemoviridae*, *Tombusviridae*, *Hepeviridae*, and *Caliciviridae* (Supplementary Fig. 8). We previously proposed that the organizations of capsid proteins might explain the evolution linkage according to the number of linkers and positions of protrusions^13^. The results indicate that the shrimp nodaviruses show structural similarity with viruses from family *Tombusviridae* greater than that with viruses from alpha and betanodaviruses, as well as families *Hepeviridae* and *Caliciviridae*, suggesting that the structural evolutionary of the shrimp nodaviruses might lie between the betanodvirus and *Sebemoviridae*.

The structural insights of the shrimp nodaviruses derived from our study provide opportunities for the rational design of antivirals, such as a recombinant MrNV-LP into *M. rosenbergii* as a potential vaccine against white-tail disease^40^. The noninfectious empty particles may have a potential as vaccine candidates. MrNV has been indicated as an effective candidate for the delivery of plasmid DNA because of its resistance to enzyme digestion^41^. The assembly to control the size distribution of shrimp nodavirus capsids with various factors discussed here might provide a harnessed platform for the delivery of therapeutic agents for biomedical applications against harsh conditions.

## Methods

### Plasmid construction and protein expression and purification

The synthetic genes of PvNV capsid protein (encoding residues 1–368), PvNVPd (encoding residues 250–368), ΔN-ARM-PvNVSd (encoding residues 38–250), ΔN-ARM-PvNV (encoding residues 45–368), and MrNVPd (encoding residues 246–371) were PCR-amplified from PvNV capsid protein (GenBank accession No. ABO33432.2) and MrNV capsid protein (GenBank accession No. ACY26145.1), respectively, and then subcloned into the artificial vector between the *Sfo*I and *Xho*I restriction sites to produce the *N*-terminal hexa-histidine-SUMO-tagged fusion proteins, as described previously^13^ (Table 1). The PvNV capsid protein was also cloned into the vector pET21a (Table 1). These fusion recombinant capsid proteins, including the truncated capsid proteins, were expressed in *Escherichia coli* BL21-CodonPlus(*DE3*)-RIL strain and were induced by isopropyl-β-_D_-thiogalactopyranoside (IPTG, Bioshop) (final concentration 0.4 mM) at 18 °C overnight. The cells were harvested by centrifugation (7000×*g*) for 20 min and were subsequently lysed by sonication in a binding buffer containing Tris-HCl (50 mM, pH 8.0), NaCl (0.25 M), imidazole (20 mM), β-mercaptoethanol (5 mM), and EGTA (1 mM). The lysate was clarified by centrifugation (10,000×*g*) for 30 min and was then filtered through a filter (0.22 μm). All viral capsid proteins were purified by nickel-nitrilotriacetic acid affinity chromatography through an IMAC column (GE Healthcare). The affinity column was washed with 4-column volumes of binding buffer supplemented with imidazole (10–50 mM); and the capsid proteins were eluted with a linear gradient of imidazole (100–500 mM). Finally, the *N*-terminal SUMO fusion tag was cleaved off with SUMO protease and removed with an IMAC column. The purified truncated capsid proteins (0.3 mg mL^−1^) were further exchanged into the final buffer containing HEPES (50 mM, pH 7.4) and NaCl (300 mM), and eventually concentrated to ~20 mg mL^−1^ for crystallization.

The purified PvNV capsid proteins obtained from SUMO or pET21a vectors were dialyzed into a buffer with CaCl_2_ (5 mM) for particle assembly. Under SDS denaturing conditions, the dimer form of PvNV capsid proteins showed a major band on SDS-PAGE corresponding to a molecular mass ~72 kDa. Size determination of PvNV-LP measured by size-exclusion chromatography (Superose 6 10/300 GL column, GE Healthcare) showed a molecular mass corresponding to the *T* = 3 capsid. The morphologies of the PvNV-LP and pET21-PvNV-LP observed with negative-staining EM revealed a particle polymorphism with diameters 29–35 and 22–24 nm consistent with *T* = 3 and *T* = 1 architectures, respectively.

To generate seleno-methionine (Se-Met) labeled PvNVPd and MrNVPd, transformed *E. coli* BL21(*DE3*) was grown in a M9 minimal medium supplemented with specific amino acids and Se-Met. The Se-Met substituted proteins were purified by procedures similar to those described above.

For structural characterizations of the PvNV, we followed the experimental procedures summarized in the flow chart given in Supplementary Fig. 1.

### Electron microscopy

For cryo-EM experiments, the sample solution (3 μL, concentration 6 mg mL^−1^) containing the *T* = 3 and *T* = 1 PvNV-LPs and the pET21-PvNV-LP was applied to a Quantifoil holey carbon grid (R1.2/1.3, Mo 200 mesh, Quantifoil Micro Tools GmbH) at 4 °C with 100% humidity, and then plunge-frozen into liquid ethane using a Vitrobot mark IV (Thermo Fisher Scientific). The cryo-EM grids were examined at a liquid-nitrogen temperature with a cryo-electron microscope (Titan Krios, Thermo Fisher Scientific), incorporating a field emission gun and a Cs-corrector (Corrected electron optical systems GmbH). The microscope was operated at 300 kV and a nominal magnification of 59,000×. Movies were recorded using a Falcon 3EC direct electron detector in a linear mode (Thermo Fisher Scientific), applied with a nominal underfocus value ranging from −1.25 to −2.75 μm. An accumulated dose of 40 electrons per Å^2^ on the sample was fractionated into a movie stack of 26 image frames with 0.077 s per frame, for a total exposure time 2.0 s. The movies (1.12 Å/pixel) were subsequently aligned and summed using the *MotionCor2* software^42^ to obtain a final dose weighted image. Estimation of the contrast transfer function was performed using the *CTFFIND* program^43^. Micrographs exhibiting poor power spectra (based on the extent and regularity of the Thong rings) were rejected. Large *T* = 3 PvNV-LPs of ~29–35 nm in diameter and small *T* = 1 PvNV-LPs of ~22–24 nm in diameter were clearly distinguished by their sizes in the cryo-EM images (Fig. 6a), and they were separately selected in *RELIOIN 2.1*^44,45^. Particles of these two types were partially separated from each other on size-exclusion chromatography with a Superose 6 10/300 GL column (Fig. 6b). All subsequent processing steps were performed in *RELION 2.1*^44,45^ (Supplementary Fig. 2). Initially, approximately 1000–2000 particles for each *T* = 3 and *T* = 1 PvNV-LP were manually picked and subjected to reference-free two-dimensional (2D) classification. Representative 2D-class averages were selected as templates for automated particle picking (Supplementary Fig. 3b). All the picked particles (*T* = 3: 42,751 particles, *T* = 1: 476,834 particles) were subjected to reference-free 2D classification and then to three-dimensional (3D) classification with icosahedral symmetry. The density maps of *T* = 3 MrNV (EMD3655) at a resolution of 60 Å and *T* = 1 Hepatitis E virus (PDB 2ZZQ) at a resolution of 40 Å were used as the initial alignment references for 3D structure refinement and post-processing. The resolutions of the *T* = 3 and *T* = 1 PvNV-LPs were estimated to be 3.5 and 3.7 Å by the gold-standard Fourier shell correlation (FSC = 0.143 criterion), respectively, after applying a soft spherical mask on the two reconstructions refined from the half of the data sets independently. The work flow of the cryo-EM single particle reconstruction was shown in Supplementary Fig. 2.

A 50 μg mL^−1^ of the diluted sample was placed onto the glow discharged carbon-coated grid for 1 min, and then the filter paper was used to blot away the excess of the buffer. After that, the sample on the grid was then washed once using ddH_2_O followed by blotting. The uranyl acetate (2% w/v) was used to stain the sample. The sample on the grid was left for air-dry before TEM observation. The sample were imaged using JEM1400 at 120 keV and recorded by Gatan Ultrascan 4000 CCD-camera model 895 (4k × 4k) (Gatan Inc., USA) at a magnification of 60,000×.

### Protein crystallization

Crystallization trials were performed with the hanging-drop vapor-diffusion method by a liquid-handling robot (Mosquito, TTP Labtech) for high-throughput screening at 18 °C. Native and Se-Met-derivatized crystals of PvNVPd and MrNVPd were grown under varied crystallization conditions. The native crystals of PvNVPd and MrNVPd were obtained from conditions consisting of Na cacodylate (0.1 M, pH 6.5), MgCl_2_ (0.075 M), PEG2000 MME (30% w/v), and MES (0.1 M, pH 6.5), MgSO_4_ (1.6 M), respectively. Se-Met-derivatized crystals were crystallized in one condition with MES (0.1 M, pH 6.5), PEG4000 (30% w/v) for Se-PvNVPd; and two conditions consisting of HEPES (0.1 M, pH 7.5), Na acetate (1.0 M), cadmium sulfate 8/3-hydrate (50 mM), and MES (0.1 M, pH 6.5), Zn acetate (0.2 M), PEG8000 (10% w/v) for Se-MrNVPd, respectively. The ΔN-ARM-PvNVSd crystals were grown in a well solution containing Tris (0.1 M, pH 8.0) and Poly (acrylic acid sodium salt) 5100 (20% v/v). The crystals reached full size in 10–14 days. These conditions were further optimized to improve the diffraction quality and resolution. The crystals were transferred from a crystallization drop into cryoprotectant solution (2 μL) with precipitant solution containing glycerol (25–30% v/v) for a few seconds, mounted on a synthetic nylon loop (0.1–0.2 mm; Hampton Research) and then flash-cooled in liquid nitrogen.

### X-ray diffraction data and structure determination

X-ray diffraction data of native crystals including PvNVPd, MrNVPd, and ΔN-ARM-PvNVSd were collected at beamline BL44XU at SPring-8 (Harima, Japan) with a CCD detector (MX300-HE); and on Taiwan Light Source beamlines 13C1 and 15A1 and Taiwan Photon Source 05A at NSRRC (Hsinchu, Taiwan) with Q315r, MX300-HE and MX300-HS CCD detectors, respectively. The native data sets were indexed, integrated and scaled by *HKL2000*^46^. Se-Met data on the PvNVPd and MrNVPd crystals were collected at the same synchrotron beamlines and processed as above.

The phases of PvNVPd and MrNVPd were determined by the SAD method using data collected from each of the Se-Met crystals at resolution 1.28 and 1.39 Å, respectively. The initial models of the PvNVPd and MrNVPd with each SAD dataset were determined using *Phenix AutoSol*^47^, with overall figures of merit 0.51 and 0.49, respectively. All the final structures of the PvNVPd and MrNVPd with varied space groups and resolutions were subsequently refined with SAD models to reliable *R*_work_/*R*_free_ values via iterative rounds of model building and refinement using *Coot*^48^ and *Refmac5*^49^.

X-ray diffraction data to 3.1 Å were collected at 100 K on the ΔN-ARM-PvNVSd crystals. The protein was crystallized in space group *P*2_1_2_1_2_1_ with one *T* = 1 particle of the ΔN-ARM-PvNVSd in the asymmetric unit, and the solvent content was 56%. The structure of ΔN-ARM-PvNVSd was determined with molecular replacement using our previously determined structure of *T* = 1 GNNV SVP (PDB 4RFT) as the search model^13^. Residues 66–249 were manually traced into the electron density corresponding to the ΔN-ARM-PvNVSd structure. The poor quality of the electron density from residues 38–65 resulted from the flexibility of the *N*-terminal region hanging in the inner cavity. The final model was refined to *R*/*R*_free_ values 0.219/0.221 under the condition of noncrystallographic-symmetry (or icosahedral) constraints using *Coot*^48^ and *Refmac5*^49^.

### Cryo-EM structure refinement and model building

The crystal structures of the ΔN-ARM-PvNVSd and PvNVPd are both used as the initial model directly docking into the EM map. Each PvNV capsid protein was then split into two parts, an *N*-terminal S-domain and a *C*-terminal P-domain, for subsequent rigid body-fitting into these two cryo-EM density maps with *Coot*^48^. On placing these two cryo-EM density maps and these two PvNV capsid protein models into an artificial unit cell, we used the *Phenix*^47^ suite for iterative, phased refinement. The quality of the final model was analyzed using *Molprobity*^50^ (Table 2). All figures of cryo-EM and crystal structures and molecular surface calculations were prepared with *PyMol* (http:// www.pymol.org).

### Reporting summary

Further information on experimental design is available in the Nature Research Reporting Summary linked to this article.

## Supplementary information

Supplementary Information

Reporting Summary

## Data Availability

Coordinates for the cryo-EM and crystal structures of PvNV and MrNV are deposited in the Protein Data Bank under accession codes 6AB5, 6AB6, 5YKU, 5YKV, 5YKX, 5YKZ, 5YL0, and 5YL1. Cryo-EM reconstructions of the *T* = 3 and *T* = 1 PvNV-LPs are deposited in the EM Data Bank under accession codes EMD-9576 and EMD-6999, respectively. All relevant data supporting the findings of this study are available from the authors upon request.

## References

[1] Araneda M, Perez EP, Gasca-Leyva E (2008). White shrimp Penaeus vannamei culture in freshwater at three densities: condition state based on length and weight. Aquaculture.

[2] Hameed ASS, Bonami JR (2012). White tail disease of freshwater prawn, *Macrobrachium rosenbergii*. Indian. J. Virol..

[3] Arcier JM (1999). A viral disease associated with mortalities in hatchery-reared postlarvae of the giant freshwater prawn *Macrobrachium rosenbergii*. Dis. Aquat. Organ..

[4] Walker PJ, Winton JR (2010). Emerging viral diseases of fish and shrimp. Vet. Res..

[5] Qian D (2003). Extra small virus-like particles (XSV) and nodavirus associated with whitish muscle disease in the giant freshwater prawn, *Macrobrachium rosenbergii*. J. Fish. Dis..

[6] Tang KFJ, Pantoja CR, Redman RM, Lightner DV (2007). Development of in situ hybridization and RT-PCR assay for the detection of a nodavirus (PvNV) that causes muscle necrosis in *Penaeus vannamei*. Dis. Aquat. Organ..

[7] Flegel TW (2012). Historic emergence, impact and current status of shrimp pathogens in Asia. J. Invertebr. Pathol..

[8] Widada J (2003). Genome-based detection methods of *Macrobrachium rosenbergii* nodavirus, a pathogen of the giant freshwater prawn, *Macrobrachium rosenbergii*: dot-blot, in situ hybridization and RT-PCR. J. Fish. Dis..

[9] Senapin S (2012). Infections of MrNV (*Macrobrachium rosenbergii* nodavirus) in cultivated whiteleg shrimp Penaeus vannamei in Asia. Aquaculture.

[10] Schuster S (2014). A unique nodavirus with novel features: mosinovirus expresses two subgenomic RNAs, a capsid gene of unknown origin, and a suppressor of the antiviral RNA interference pathway. J. Virol..

[11] Chao JA (2005). Dual modes of RNA-silencing suppression by flock house virus protein B2. Nat. Struct. Mol. Biol..

[12] Wang ZW (2013). Characterization of a nodavirus replicase revealed a de novo initiation mechanism of RNA synthesis and terminal nucleotidyltransferase activity. J. Biol. Chem..

[13] Chen NC (2015). Crystal Structures of a piscine betanodavirus: mechanisms of capsid assembly and viral infection. PLoS Pathog..

[14] Zhang HJ (2006). Quantitative relationship of two viruses (MrNV and XSV) in white-tail disease of *Macrobrachium rosenbergii*. Dis. Aquat. Organ..

[15] Ho KL, Kueh CL, Beh PL, Tan WS, Bhella D (2017). Cryo-electron microscopy structure of the *Macrobrachium rosenbergii* nodavirus capsid at 7 angstroms resolution. Sci. Rep..

[16] Guo Y (2014). Crystal structure of a nematode-infecting virus. Proc. Natl Acad. Sci. USA.

[17] Morgunova E (1994). The atomic-structure of carnation mottle virus capsid protein. FEBS Lett..

[18] Li M (2013). Atomic structure of cucumber necrosis virus and the role of the capsid in vector transmission. J. Virol..

[19] Satheshkumar P (2004). Role of metal ion-mediated interactions in the assembly and stability of Sesbania mosaic virus *T* = 3 and *T* = 1 capsids. J. Mol. Biol..

[20] Banerjee M (2010). Structure and function of a genetically engineered mimic of a nonenveloped virus entry intermediate. J. Virol..

[21] Belyi VA, Muthukumar M (2006). Electrostatic origin of the genome packing in viruses. Proc. Natl Acad. Sci. USA.

[22] Qu C (2000). 3D domain swapping modulates the stability of members of an icosahedral virus group. Structure.

[23] Hopper P, Harrison SC, Sauer RT (1984). Structure of tomato bushy stunt virus. V. Coat protein sequence determination and its structural implications. J. Mol. Biol..

[24] Guu TSY (2009). Structure of the hepatitis E virus-like particle suggests mechanisms for virus assembly and receptor binding. Proc. Natl Acad. Sci. USA.

[25] Yamashita T (2009). Biological and immunological characteristics of hepatitis E virus-like particles based on the crystal structure. Proc. Natl Acad. Sci. USA.

[26] Zheng H (2017). CheckMyMetal: a macromolecular metal-binding validation tool. Acta Crystallogr. D Biol. Crystallogr..

[27] Tang JH (2006). The role of subunit hinges and molecular “switches” in the control of viral capsid polymorphism. J. Struct. Biol..

[28] Sangita V (2004). *T* = 1 capsid structures of Sesbania mosaic virus coat protein mutants: determinants of *T* = 3 and *T* = 1 capsid assembly. J. Mol. Biol..

[29] Hsu C (2006). Characterization of polymorphism displayed by the coat protein mutants of tomato bushy stunt virus. Virology.

[30] Kakani K, Reade R, Katpally U, Smith T, Rochon D (2008). Induction of particle polymorphism by Cucumber Necrosis Virus coat protein mutants in vivo. J. Virol..

[31] Niu S (2014). Hibiscus chlorotic ringspot virus coat protein is essential for cell-to-cell and long-distance movement but not for viral RNA replication. Plos One.

[32] Li SW (2009). Dimerization of hepatitis e virus capsid protein E2s domain is essential for virus-host interaction. PLoS Pathog..

[33] Liu W (2015). A unique human norovirus lineage with a distinct HBGA binding interface. PLoS Pathog..

[34] Somrit M (2017). C-terminal domain on the outer surface of the *Macrobrachium rosenbergii* nodavirus capsid is required for Sf9 cell binding and internalization. Virus Res..

[35] Ito Y (2008). Variable region of betanodavirus RNA2 is sufficient to determine host specificity. Dis. Aquat. Organ..

[36] Kalynych S (2016). Virion structure of iflavirus slow bee paralysis virus at 2.6-angstrom resolution. J. Virol..

[37] Kalynych S, Fuzik T, Pridal A, de Miranda J, Plevka P (2017). Cryo-EM study of slow bee paralysis virus at low pH reveals iflavirus genome release mechanism. Proc. Natl Acad. Sci. USA.

[38] Dokland T (2000). Freedom and restraint: themes in virus capsid assembly. Struct. Fold. Des..

[39] Hanapi UF (2017). Tracking the virus-like particles of *Macrobrachium rosenbergii* nodavirus in insect cells. PeerJ.

[40] Farook M (2014). Immunomodulatory effect of recombinant *Macrobrachium rosenbergii* nodavirus capsid protein (r-MCP) against white tail disease of giant freshwater prawn, *Macrobrachium rosenbergii* (de Man, 1879). Aquaculture.

[41] Jariyapong P (2014). Encapsulation and delivery of plasmid DNA by virus-like nanoparticles engineered from *Macrobrachium rosenbergii* nodavirus. Virus Res..

[42] Zheng SQ (2017). MotionCor2: anisotropic correction of beam-induced motion for improved cryo-electron microscopy. Nat. Methods.

[43] Rohou A, Grigorieff N (2015). CTFFIND4: fast and accurate defocus estimation from electron micrographs. J. Struct. Biol..

[44] Scheres SHW (2012). RELION: Implementation of a Bayesian approach to cryo-EM structure determination. J. Struct. Biol..

[45] He S, Scheres SHW (2017). Helical reconstruction in RELION. J. Struct. Biol..

[46] Otwinowski Z, Minor W (1997). Processing of X-ray diffraction data collected in oscillation mode. Method Enzymol..

[47] Adams PD (2010). PHENIX: a comprehensive Python-based system for macromolecular structure solution. Acta Crystallogr. D Biol. Crystallogr..

[48] Emsley P, Lohkamp B, Scott WG, Cowtan K (2010). Features and development of Coot. Acta Crystallogr. D Biol. Crystallogr..

[49] Murshudov GN (2011). REFMAC5 for the refinement of macromolecular crystal structures. Acta Crystallogr. D Biol. Crystallogr..

[50] Chen VB (2010). MolProbity: all-atom structure validation for macromolecular crystallography. Acta Crystallogr. D Biol. Crystallogr..

