# Supplementary material for: The atomic structures of shrimp nodaviruses reveal new dimeric spike structures and particle polymorphism
Source: Commun Biol. 2019 Feb 20;2:72. doi: 10.1038/s42003-019-0311-z (PMC6382870; doi:10.1038/s42003-019-0311-z)
Supplement: Supplementary file 1 — Supplementary Information [file 42003_2019_311_MOESM1_ESM.pdf]

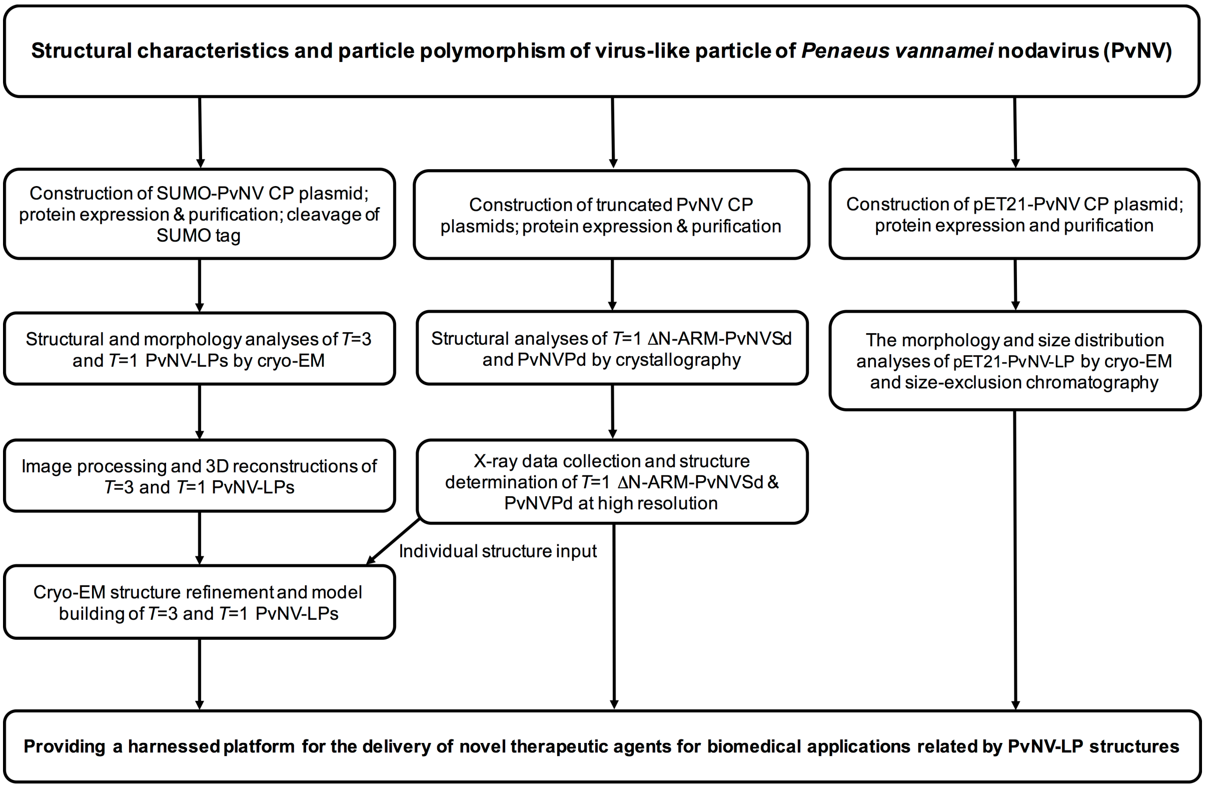

Supplementary Figure 1. The overall flow chart for the structural characterizations of the PvNV.

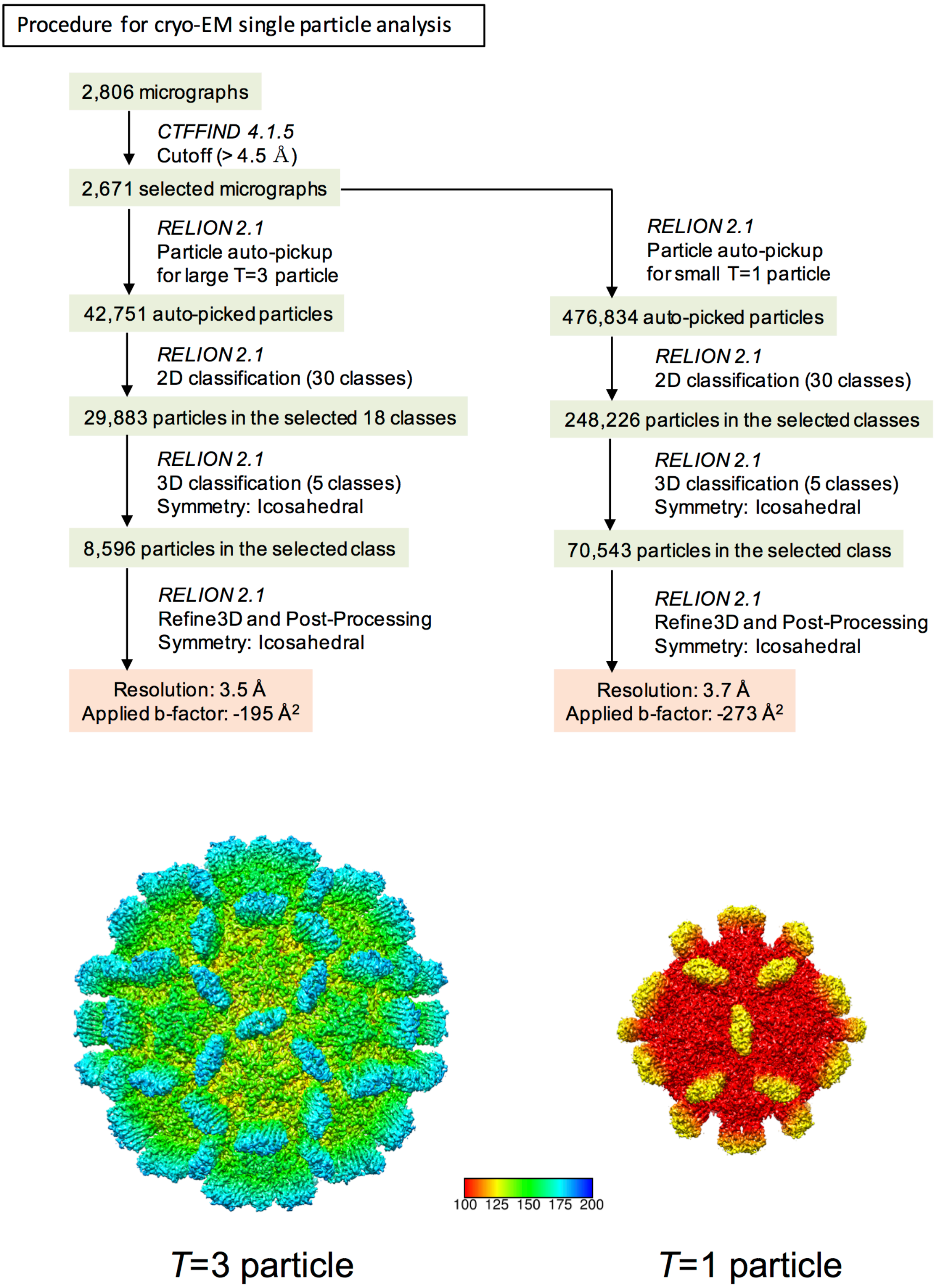

Supplementary Figure 2. The procedure for the analysis of cryo-EM images of single particles of *T*=3 and *T*=1 PvNV-LPs.

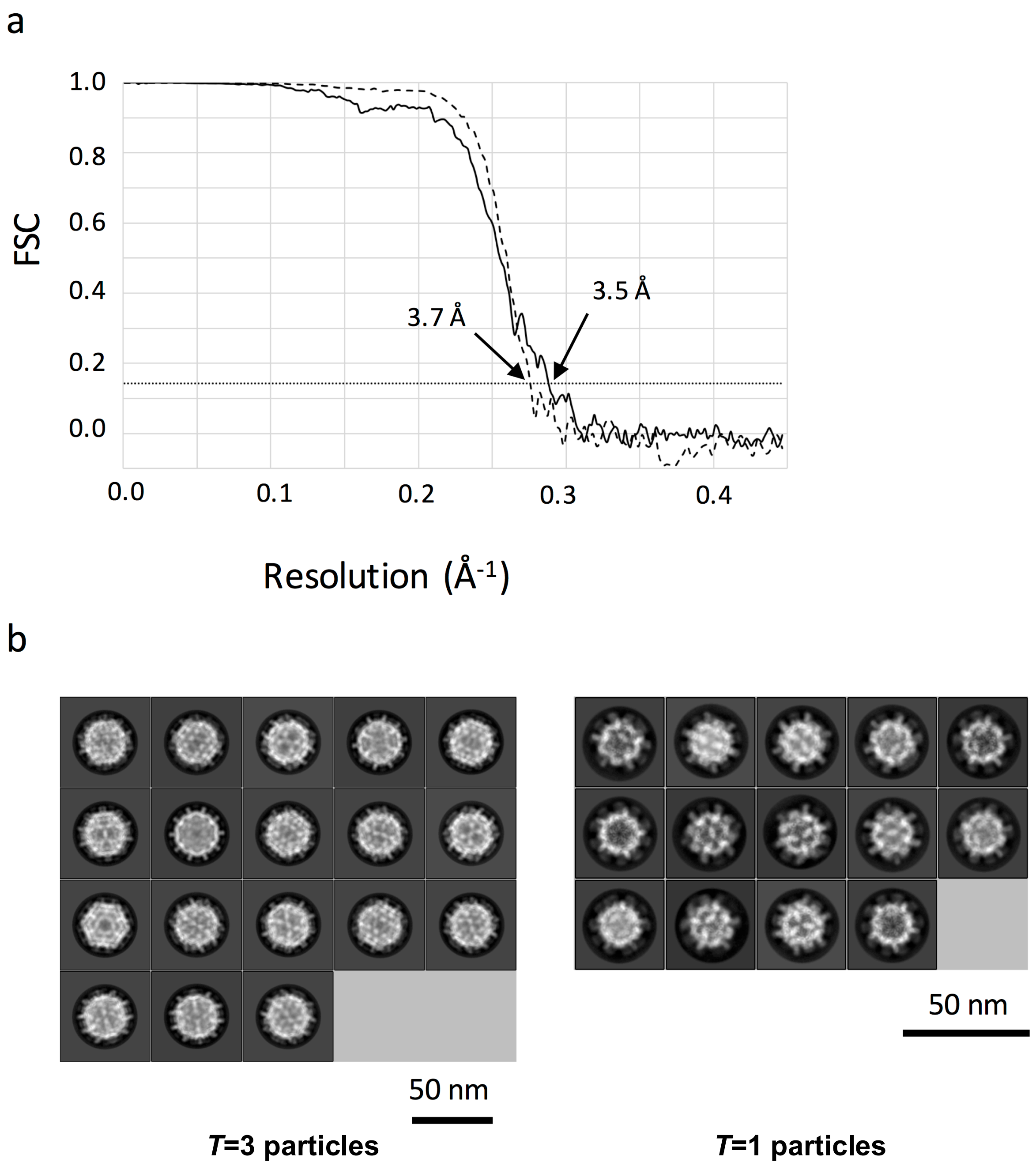

Supplementary Figure 3. Characterization of the 3D density map and 2D class-averages. (**a**) Fourier shell correlation (FSC) curves of the 3D reconstructions of *T*=1 (*dashed line*) and *T*=3 (*solid line*) PvNV-LPs. (**b**) Representative 2D class-average images (Scale bars: 50 nm).

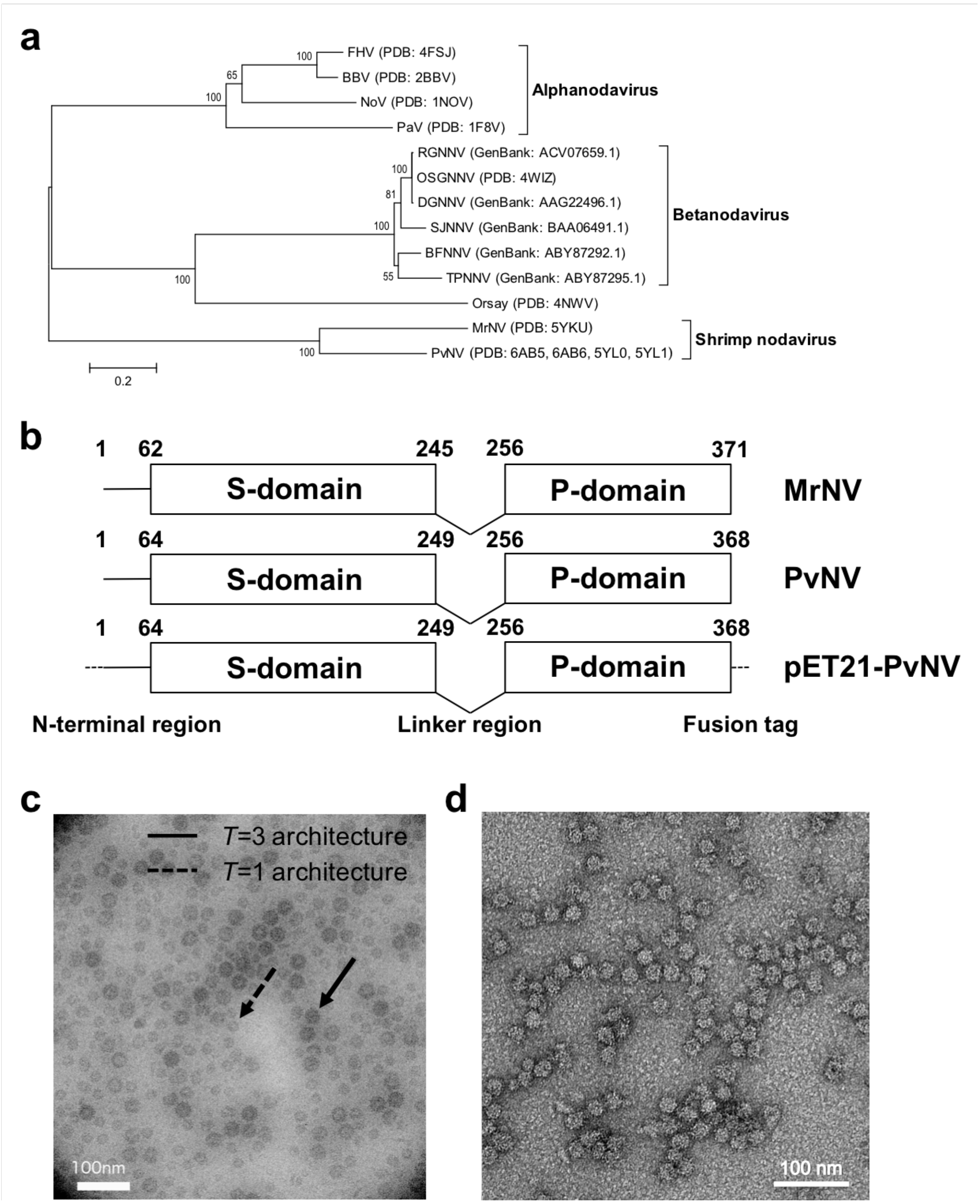

Supplementary Figure 4. Phylogenetic tree of CPs of the family *Nodaviridae*. (**a**) The phylogenetic tree of the family *Nodaviridae*. The neighbor-joining phylogenetic unrooted tree was built using Mega5 with a multiple alignment of conserved blocks of the sequence of RNA2. The major clades of the family *Nodaviridae* are identified as alphanodavirus, betanodavirus and shrimp nodavirus, respectively. (**b**) Schematic diagrams showing the domain structures of MrNV, PvNV and pET21-PvNV in this study. The *N*-terminal region, S-domain, linker region, P-domain and fusion tags are indicated. (**c**) The EM image of the pET21-PvNV-LP. The

different diameters of the pET21-PvNV-LPs correspond to the *T*=3 and *T*=1 icosahedral particles. (**d**) The EM image of the ∆N-ARM-PvNV. The diameter of the ∆N-ARM-PvNV corresponds to *T*=1 icosahedral particle.

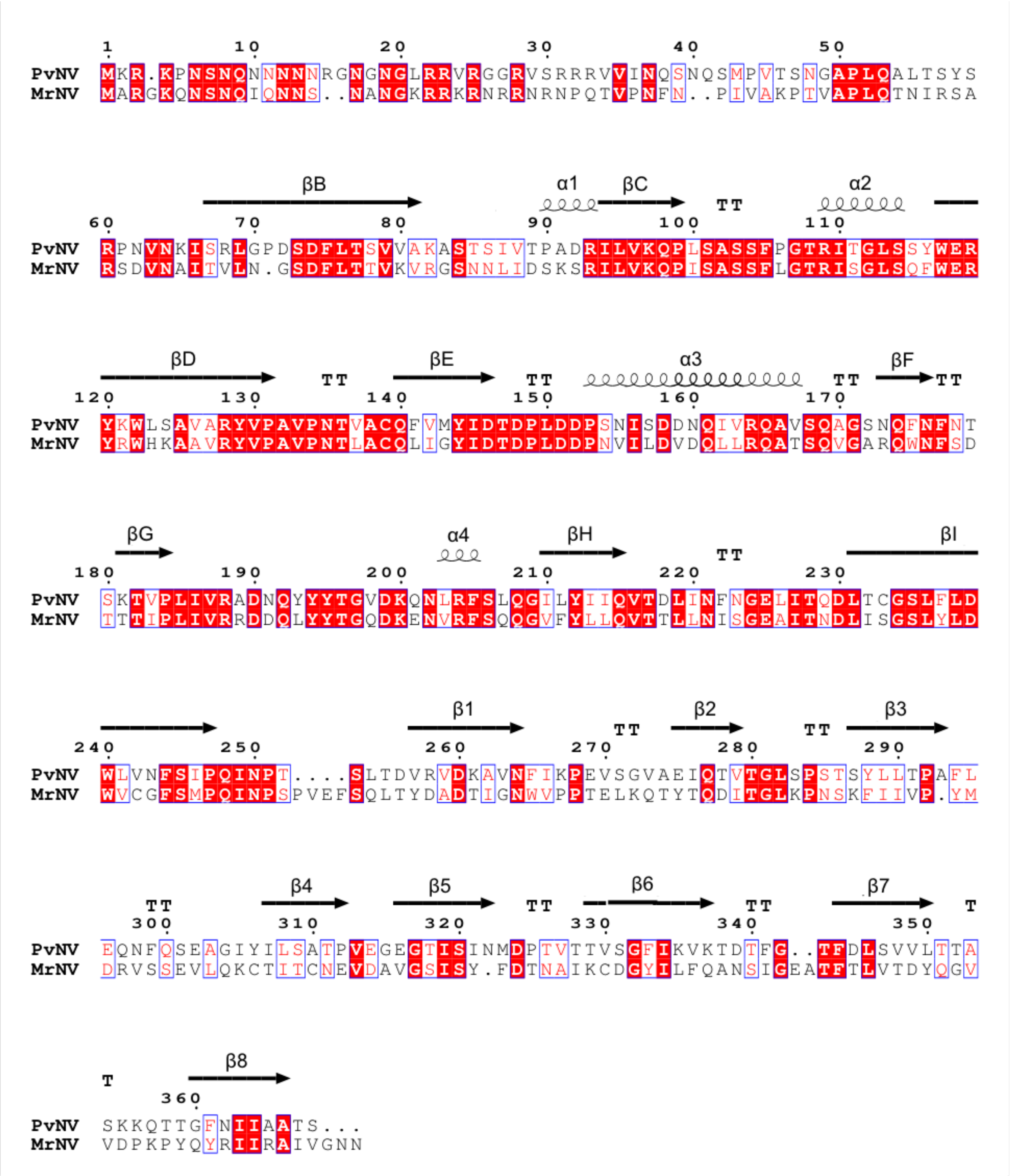

Supplementary Figure 5. The sequence alignment of RNA2-encoded CPs from two strains of shrimp nodavirus. Multiple sequence alignment was performed by ClustalW. The secondary structure of shrimp nodavirus CP is shown above the sequence. Fully conserved residues are shaded in red.

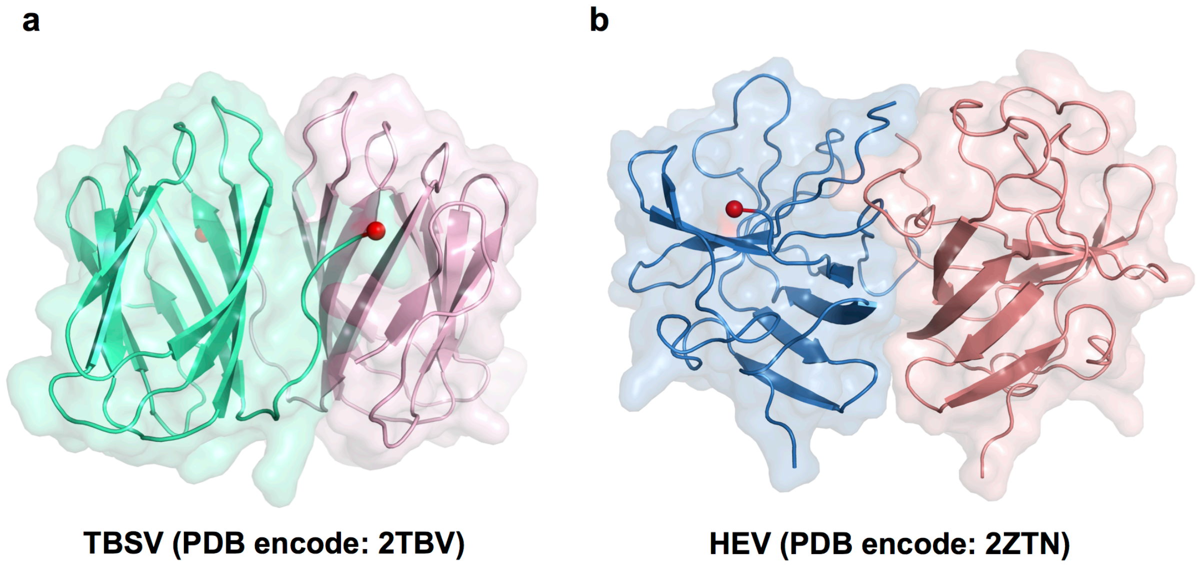

Supplementary Figure 6. A comparison with an alignment of the P-domains from two different representative viruses: TBSV and HEV. (**a, b**) The P-domain and P2-domain from TBSV and HEV are colored in *green*, *pink*, *blue* and *salmon*, respectively. The *C*-terminus is colored as a red sphere.

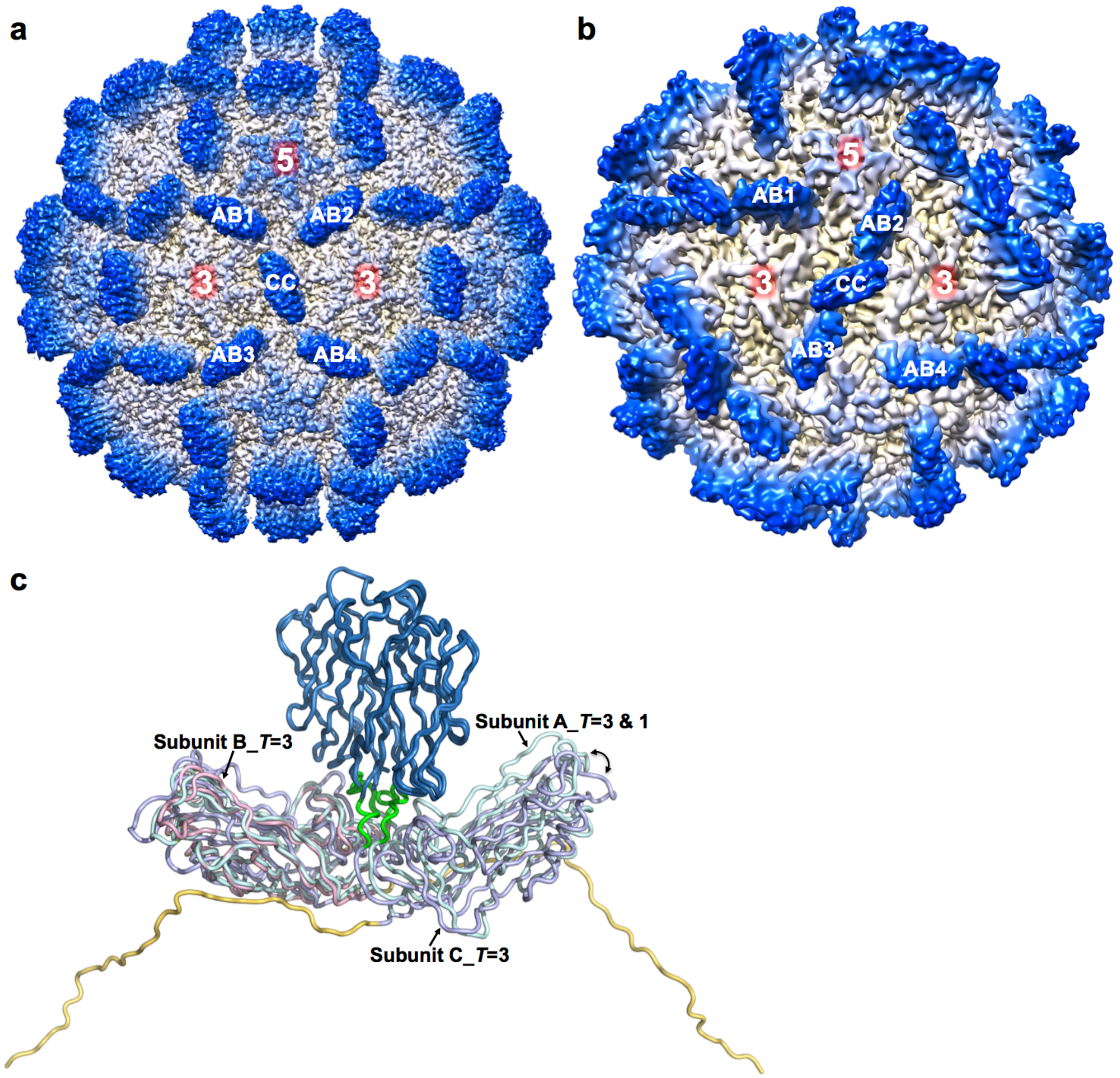

Supplementary Figure 7. The comparison of dimer spikes between *T*=3 PvNV-LP and *T*=3 MrNV-LP. The cryo-EM structures for *T*=3 PvNV-LP (**a**) and *T*=3 MrVN-LP (**b**) comprise A/B and C/C dimeric P-domains arranged alternately around the I2, I3 and I5 axes. (**c**) Superposition of the Cα backbone of the subunit-A/B and C/C dimers from *T*=3 PvNV-LP and subunit-A/A dimer from *T*=1 PvNV-LP. The P-domain, linker region and N-arm are shown in *blue*, *green* and *yellow*, respectively. The S-domain from three subunits A, B, and C are shown in *light green*, *pink* and *purple*, respectively.

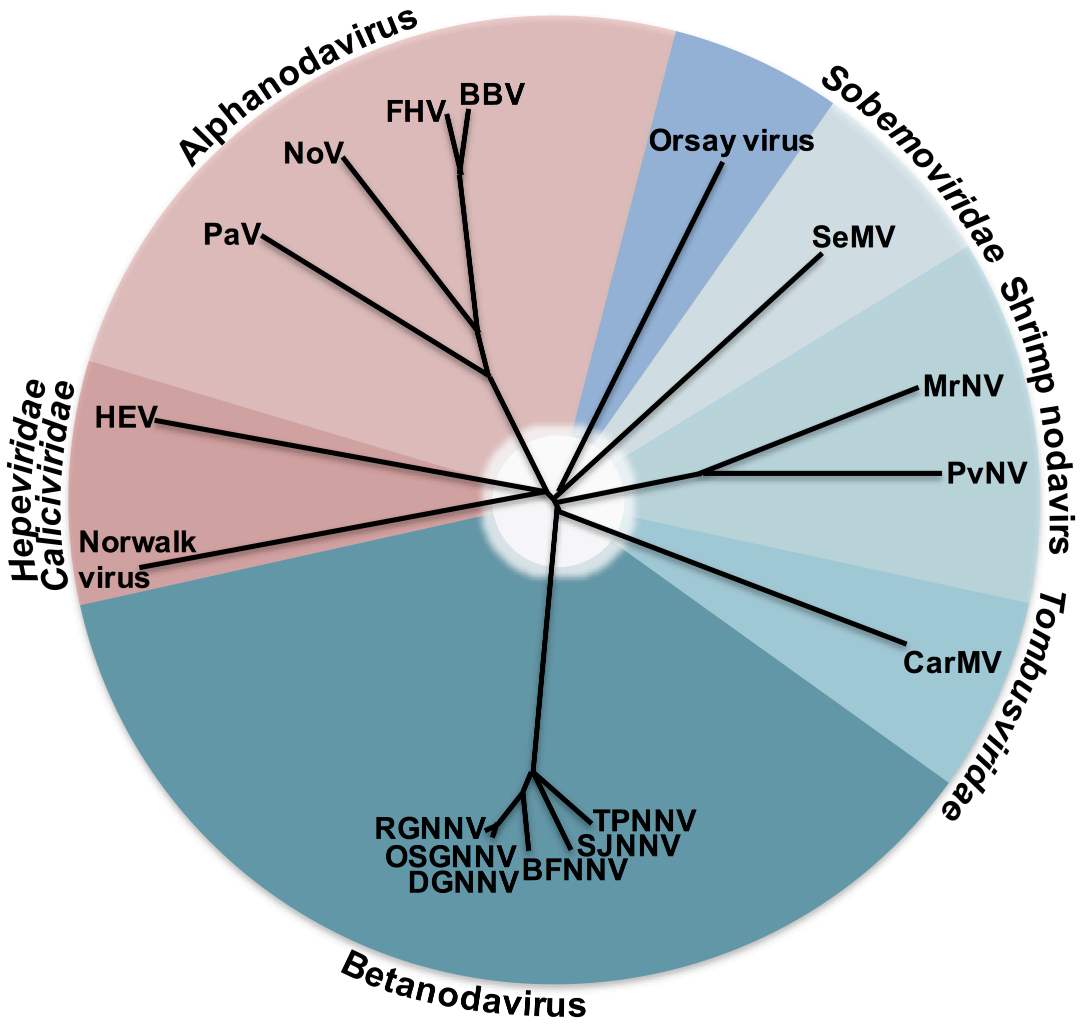

Supplementary Figure 8. Evolutionary relationship among viruses from the families *Nodaviridae*, *Sebemoviridae*, *Tombusviridae*, *Hepeviridae* and *Caliciviridae* based on structural alignment of their CPs by SplitsTree[1]. Shown are representative viral CPs from different viruses, including nodamura virus (NoV; PDB 1NOV), black beetle virus (BBV; PDB 2BBV), pariacoto virus (PaV; PDB 1F8V), flock house virus (FHV; PDB 4FSJ), striped jack nervous necrosis virus (SJNNV), tiger puffer nervous necrosis virus (TPNNV), red-spotted grouper nervous necrosis virus (RGNNV), orange-spotted grouper nervous necrosis virus (OSGNNV; PDB 4WIZ), dragon grouper nervous necrosis virus (DGNNV), barfin flounder nervous necrosis virus (BFNNV), orsay virus (PDB 4NWV), carnation mottle virus (CarMV; PDB 1OPO), HEV (PDB 2ZTN and 3HAG), norwalk virus (PDB 1IHM), PvNV and MrNV, respectively.

Supplementary Reference

1. Huson, D.H. SplitsTree: analyzing and visualizing evolutionary data. *Bioinformatics* **14**, 68–73 (1998).
